# A network meta-analysis of mind–body exercise interventions for internet addiction symptoms in young adults

**DOI:** 10.3389/fpubh.2025.1565372

**Published:** 2025-06-18

**Authors:** Shiguan Jia, Zhihao Du, Dengshan Chu, Jiayi Yao, Haozhe Wang, Wenjia Chen, Dazhong Zhang, Xin Zang

**Affiliations:** ^1^School of Physical Education, China University of Mining and Technology, Xuzhou, Jiangsu, China; ^2^Department of Physical Education, Dankook University, Yongin, Republic of Korea

**Keywords:** mind–body exercise, young adults, internet addiction, intervention effectiveness, network meta-analysis

## Abstract

**Background:**

Internet Addiction Disorder (IAD) is a recurrent mental illness. It severely impacts both physical and mental health, leading to physiological symptoms such as neurological dysfunction and immunosuppression. This condition significantly affects adolescent development and has become a global public health concern. Scientific evidence supports the beneficial impact of integrative mind–body exercises for treating internet addiction disorders. Though these interventions show promise, their therapeutic efficacy exhibits considerable variation across different modalities. Currently, there exists a significant research gap, as no comprehensive clinical investigations have systematically evaluated the comparative therapeutic outcomes of distinct mind–body practices among individuals diagnosed with internet addiction.

**Objective:**

To conduct a network meta-analytic investigation comparing the therapeutic efficacy of diverse mind–body exercise interventions for addressing symptoms associated with internet addiction disorder.

**Methods:**

Data were retrieved from Web of Science, PubMed, CNKI, and VIP databases. After screening and data extraction, network meta-analysis was performed using STATA 18.0.

**Results:**

Twenty-four studies were included in the analysis. Compared with the control group (Placebo), Mindfulness [*SMD* = 13.33, *95%CI* (7.42,19.25), *p* < 0.05] and Taichi [*SMD* = −10.91, *95%CI* (−18.71,−3.11), *p* < 0.05] significantly improved internet addiction symptoms. According to *SUCRA* values, the interventions were ranked in order of effectiveness: Mindfulness (*SUCRA* = 76.3%), DanceSport (*SUCRA* = 64.0%), Yoga (*SUCRA* = 63.1%), and Taichi (*SUCRA* = 62.6%).

**Conclusion:**

Mindfulness and Tai Chi significantly reduce and alleviate internet addiction symptoms compared to Placebo. Based on SUCRA rankings, Mindfulness, Dance Sport, Yoga, and Tai Chi show the highest probability of effectiveness in descending order, providing promising options for managing internet addiction symptoms in young adults.

**Systematic Review Registration:**

https://www.crd.york.ac.uk/PROSPERO/view/CRD42025631096.

## Introduction

As “digital natives,” young adults (aged 18–24 years) represent a distinctive developmental period characterized by continued neuroplasticity and incomplete prefrontal cortex maturation, making them particularly vulnerable to Internet addiction (1–3). Neurobiological research has demonstrated that the underdevelopment of the prefrontal cortex, which is critical for impulse control and executive decision-making, combined with heightened sensitivity to social and emotional stimuli, creates a neurological landscape that predisposes university students to addictive behaviors (4–6). This critical neurodevelopmental stage features heightened reward sensitivity and still-developing inhibitory control systems, coupled with increased independence and reduced external monitoring, creating unique susceptibility to problematic Internet use behaviors (2, 7). The convergence of neurological immaturity, digital immersion, and reduced self-regulatory mechanisms significantly elevates the risk of Internet addiction among this demographic, underscoring the urgent need for targeted interventional strategies (1, 8). Internet addiction refers to compulsive Internet use behavior, characterized by an individual’s inability to control their usage, resulting in negative physiological, psychological, and social consequences (9). IAD can not only lead to physiological symptoms such as neurological dysfunction and immunosuppression (10), but also lead to psychological problems such as cognitive decline, anxiety and depression (11, 12). Olson et al.’s (13) research shows that the incidence of Internet addiction disorder in young Adults is increasing year by year, and IAD has become a major public health problem affecting the development of young Adults (14).

Current IAD interventions span a wide range, including pharmacotherapy (15), acupuncture (16), and exercise behavior (17). Growing apprehension regarding pharmaceutical adverse reactions and contraindicated drug combinations has led to heightened interest in treatment approaches that do not rely on medication (18). Research has progressively demonstrated the significant impact of physical activity on psychological well-being (19, 20). Mind–body exercise (MBE) is classified by the National Center for Complementary and Integrative Health at the U.S. National Institutes of Health as a complementary and alternative therapeutic practice (21). Mind–body exercise represents a distinctive form of physical activity that synthesizes breathing techniques, physical movements, and meditative practices (22, 23). These exercises are characterized by their low to moderate-intensity aerobic nature, featuring gentle and precise movements that emphasize the synchronization between physical actions and respiratory patterns (24). These mind–body practices combine physiological and psychological therapeutic elements (25, 26). Compared to traditional aerobic and resistance training programs, these mindfulness-based exercise practices demonstrate extraordinary practicality and contribute to sustained health improvement (27, 28). Their unique value lies in their holistic philosophy, which prioritizes the complex connections between psychological states, physiological functions, and breathing patterns. Moreover, these mind–body exercises require no special equipment (29, 30), have low learning costs, high safety levels, and are suitable for large-scale promotion across diverse populations. For patients with Internet Addiction Disorder (IAD), these comprehensive exercises provide advantages beyond traditional exercise methods, as they not only enhance physical fitness but also effectively reduce anxiety levels and improve overall quality of life. Among various forms of mind–body exercise, this study selected four therapies based on the following considerations: Representativeness; Operability; Safety; Popularity; Preliminary research foundation.

Research has indicated that integrative movement practices like Tai Chi and Dance Sport demonstrate efficacy in alleviating internet dependency symptoms (3, 31, 32). The existing literature predominantly examines therapeutic approaches in isolation (33, 34), with limited comparative studies evaluating multiple mindfulness-based movement interventions for youth internet addiction (35, 36). As highlighted in the review of Pirwani et al. (37), contemporary investigations tend to concentrate on specific demographic segments, leaving broader age-range analyses underexplored (38, 39). Network meta-analysis offers advantages over traditional meta-analytic methods by enabling both direct and indirect effect comparisons, facilitating intervention rankings (40). This investigation examined randomized controlled trials evaluating seven distinct mind–body therapeutic approaches for internet addiction disorder. By implementing network meta-analytic methodology to synthesize both direct and indirect comparative data, this research assessed relative intervention effectiveness, established comparative efficacy metrics, and generated evidence-based rankings. The findings aim to inform clinical decision-making and provide empirical foundation for young adults internet addiction treatment protocols.

This study focused on young adults aged 18–24 years, a developmental period characterized by continued neuroplasticity and heightened vulnerability to internet addiction (41, 42). While this age range extends beyond traditional adolescence, it represents a critical transition period in cognitive and behavioral development.

## Study design and methodology

Prior to initiating research, our systematic analysis protocol received formal registration through PROSPERO, an established global database for systematic review protocols (reference: CRD42025631096). This preregistration step ensured methodological transparency and adherence to standardized review practices (Table 1).

**Table 1:** PICOS-based eligibility criteria (participation, intervention comparison, outcomes, and study design).

PICOS	Criteria
Participants	Young adults
Intervention	Mind–body exercise
Comparison	Regular physical activity group
Outcome	Internet addiction
Study design	Randomized controlled trial

### Literature search

This study strictly followed the PRISMA (Preferred Reporting Items for Systematic Reviews and Meta-Analyses) guidelines (43) and established rigorous literature search, inclusion, screening, and exclusion criteria based on the PICOS (Population, Intervention, Comparison, Outcome, Study design) principle of evidence-based medicine. Boolean logic searches were conducted in Elsevier ScienceDirect, Web of Science, PubMed, ProQuest, Scopus, The Cochrane Library, CNKI, WFSDP, and VIP databases. Literature retrieval encompassed publications in both English and Chinese, with Chinese-language sources limited to prominent academic periodicals. The temporal scope of the investigation spanned from the beginning of 2010 through April 1, 2024. Supplementary relevant publications were identified through reference list examination of pertinent studies and alternative search methods. The detailed search protocol employed for the PubMed database is presented in Box 1.

BOX 1 **PubMed search strategy**

#1 (“Mind”[Title/Abstract] OR “body exercise”[Title/Abstract] OR “Mind–body exercise”[Title/Abstract] OR “Taichi”[Title/Abstract] OR “Baduanjin”[Title/Abstract] OR “Mindfulness”[Title/Abstract] OR “Dance Sport”[Title/Abstract]) OR “Qigong”[Title/Abstract] OR “Yoga”[Title/Abstract] OR “Aerobicdance”[Title/Abstract] AND “Internet Addiction Disorder”[MeSH Terms]

#2 “Sports”[Title/Abstract] OR “Sport”[Title/Abstract] OR “Athletics”[Title/Abstract] OR “Athletic”[Title/Abstract]

#3 #1 OR #2

#4 “Internet Addiction Disorder”[Title/Abstract] OR “Addiction Disorder,Internet”[Title/Abstract] OR “Addiction Disorders,Internet”[Title/Abstract] OR “Disorder,Internet Addiction”[Title/Abstract] OR “Disorders,Internet Addiction”[Title/Abstract] OR “Internet Addiction Disorders”[Title/Abstract] OR “Internet Addiction”[Title/Abstract] OR “Addiction,Internet”[Title/Abstract] OR “Addictions,Internet”[Title/Abstract] OR “Internet Addictions”[Title/Abstract] OR “Internet Gaming Disorder”[Title/Abstract] OR “Disorder,Internet Gaming”[Title/Abstract] OR “Disorders,Internet Gaming”[Title/Abstract] OR “Gaming Disorder,Internet”[Title/Abstract] OR “Gaming Disorders,Internet”[Title/Abstract] OR “Internet Gaming Disorders[Title/Abstract] OR Smartphone Addiction[Title/Abstract] OR Addiction,Smartphone[Title/Abstract] OR Addictions,Smartphone[Title/Abstract] OR Smartphone Addictions[Title/Abstract] OR Social Media Addiction[Title/Abstract] OR Addiction,Social Media[Title/Abstract] OR Addictions,Social Media[Title/Abstract] OR Media Addiction,Social[Title/Abstract] OR Media Addictions,Social[Title/Abstract] OR Social Media Addictions[Title/Abstract]

#5 “randomized controlled trial”[Publication Type] OR “controlled clinical trial”[Publication Type]

#6 #3 AND #4 AND #5 AND #6

### Inclusion criteria

The study only included randomized controlled trials (RCTs) examining mind–body exercise interventions for internet addiction. The intervention groups received one of four mind–body exercise forms (Tai Chi, Mindfulness, Baduanjin, or Dance Sport), while the control group engaged in unstructured physical activity. The outcome measure was IAD scale scores (intervention was considered effective if the difference between post-intervention and pre-intervention scores was less than 0 on any type of internet addiction scale used to assess participants’ psychological health).

### Selection restrictions

The research team eliminated investigations that met any of the following conditions: experimental designs lacking randomized control trials; research conducted on non-human subjects; qualitative analyses, questionnaire-based investigations, literature syntheses, meta-analyses, symposium abstracts, or republished materials. Additionally, we removed papers that failed to report statistical measures using mean and standard deviation notation, incorporated supplementary treatments during the research period, or presented incomplete/incompatible datasets. Research papers whose metrics could not be properly extracted were also excluded from consideration.

### Literature screening and data extraction

The evaluation process began with two independent reviewers utilizing NoteExpress V4.X to compile and manage references. After eliminating redundant entries, they assessed publication abstracts and headings. Following preliminary selection, comprehensive manuscript evaluation was conducted according to predetermined eligibility criteria. These criteria included: (1) only randomized controlled trials with human subjects; (2) studies reporting outcomes using mean and standard deviation; (3) research implementing a single intervention without supplementary treatments; (4) complete datasets with compatible metrics; and (5) studies published in peer-reviewed journals with full-text availability. When differences arose in selection outcomes, the reviewers consulted a third expert to reach consensus. Data extraction was performed independently by two team members using standardized documentation forms. The collected information encompassed: (1) Publication details (primary author identification, publication timeline, manuscript title); (2) Study population data (group sizes, demographic information including age distribution and sex ratio); (3) Methodological specifics (intervention protocols, treatment duration and intervals, Internet Addiction Disorder (IAD) measurements—specifically, baseline assessments conducted prior to intervention initiation (pre-intervention) using validated IAD diagnostic instruments such as Young’s Internet Addiction Test (IAT), Chen Internet Addiction Scale (CIAS), or other standardized tools, and follow-up assessments conducted after intervention completion (post-intervention) using identical instruments to evaluate changes in IAD severity, frequency, and associated symptoms); (4) literature quality assessment using the PEDro scale for RCTs; and (5) outcome indicators and main findings. Inter-coder reliability was 94%, with discrepancies resolved through re-checking and discussion.

### Quality assessment protocol

Two autonomous evaluators employed RevMan 5.4’s evaluation framework to systematically analyze methodological integrity. The assessment encompassed multiple domains: randomization procedures, allocation masking techniques, participant-researcher blinding protocols, outcome evaluation objectivity, data completeness, reporting transparency, and additional potential sources of systematic error. The evaluation system utilized a three-tier classification: investigations fulfilling all methodological requirements received positive marks (+) indicating minimal bias risk; those failing to meet standards were designated with negative marks (−) signifying elevated bias risk; while instances of insufficient methodological documentation were marked with uncertainty indicators (?). When evaluators reached different conclusions, they engaged a third expert reviewer to establish consensus through collaborative deliberation.

### Quantitative synthesis methods

The synthesis of data employed STATA 17.0’s networkMeta package to analyze continuous variables across studies. Given the diversity in measurement instruments, effect magnitudes were synthesized using standardized mean differences (SMD), with significance threshold established at *α* = 0.05. Statistical heterogeneity evaluation incorporated both Q and I^2^ statistics, while potential publication bias underwent examination through Egger’s regression approach. In cases involving closed-loop network configurations, inconsistency evaluation utilized node analysis techniques. When loop testing yielded *p* > 0.05, researchers proceeded with consistency model computations. Local inconsistency underwent evaluation via node-splitting methodology; instances where *p* < 0.05 prompted adherence to traditional meta-analytic direct comparison outcomes. Treatment effectiveness rankings were determined through surface under the cumulative ranking curves (SUCRA), yielding values between 0 and 1. To identify optimal therapeutic combinations, cluster analysis was performed based on these SUCRA metrics. The investigation of publication bias utilized adjusted comparison funnel plot visualization techniques.

## Research outcomes

### Document identification and selection process

**Figure 1:**
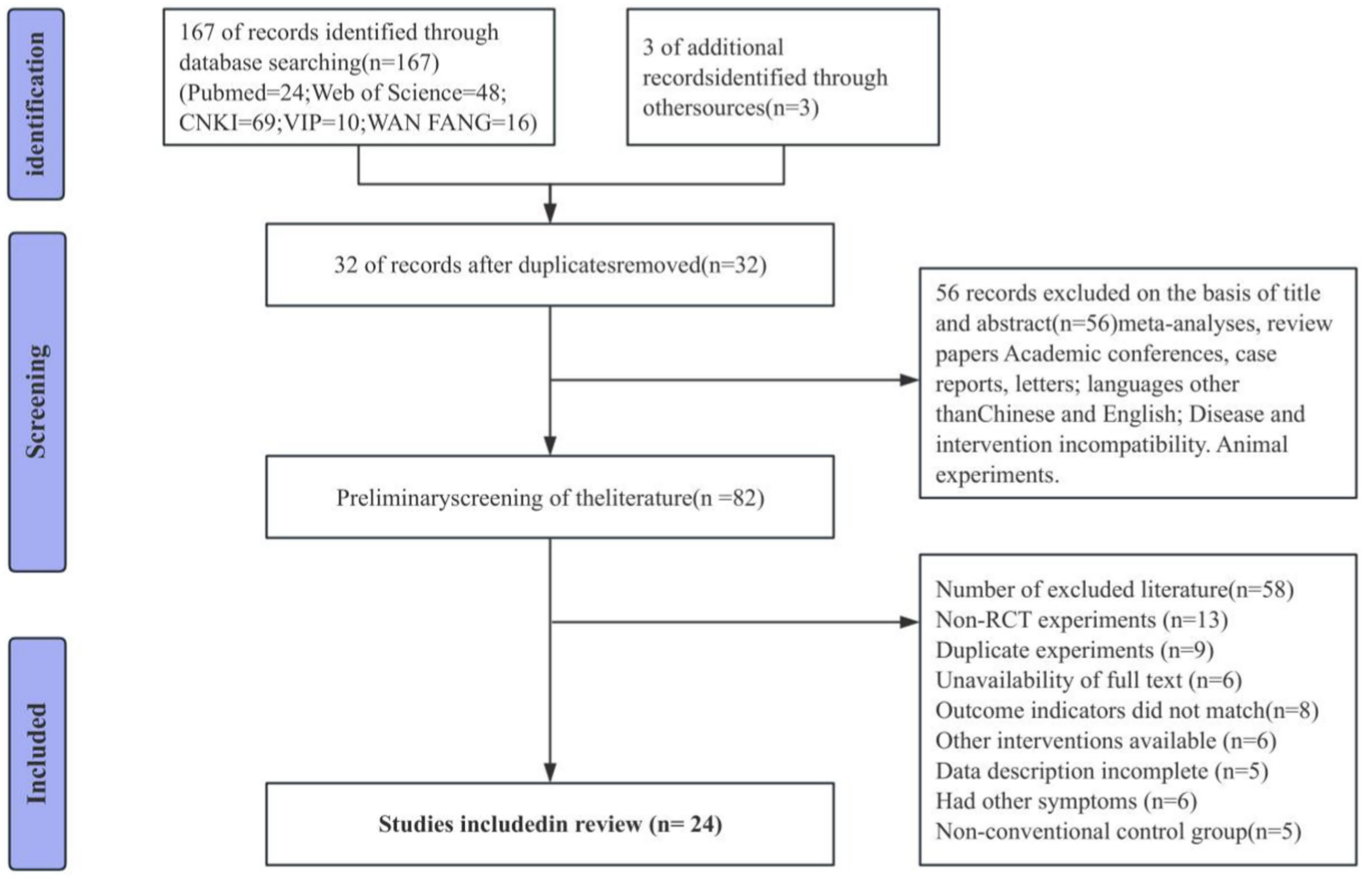
Literature screening flowchart.

The systematic search protocol yielded 170 potentially relevant publications. Database management using EndNote software, combined with manual verification, identified 32 duplicate entries, leaving 138 unique manuscripts for evaluation. Comprehensive assessment, including examination of titles, abstracts, and complete manuscripts, resulted in the final selection of 24 publications that satisfied all inclusion parameters. The selected literature includes both English and Chinese publications. A detailed visualization of the selection methodology and outcomes appears in Figure 1.

### Characteristics and quality evaluation of selected research

**Figure 2:**
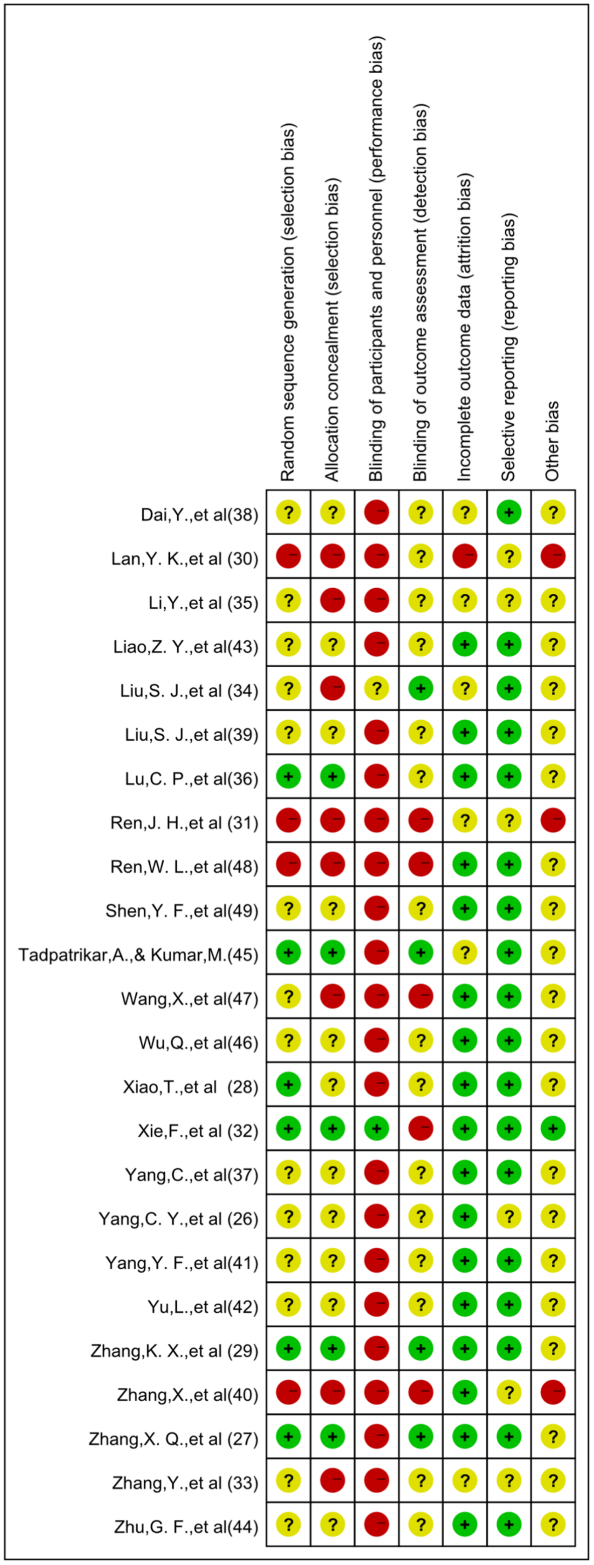
Risk of bias assessment results for included studies.

**Figure 3:**
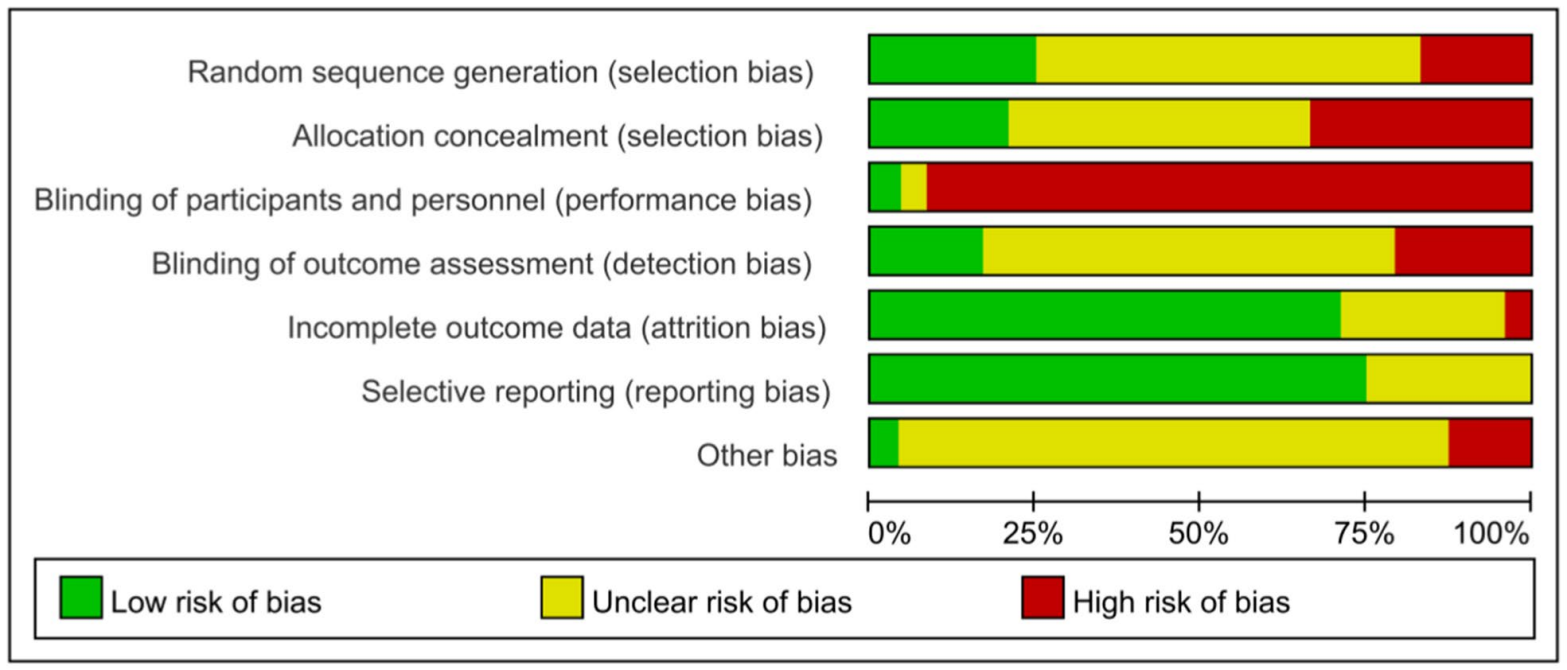
Bar graph of risk of bias assessment results for included studies.

Our systematic review synthesized findings from 24 investigations meeting inclusion parameters. Comprehensive study details appear in Table 2, encompassing research conducted on 1,711 young adults (age range: 18–24). Intervention durations spanned 8–16 weeks for measuring outcomes. Given the exercise-based nature of interventions, implementing blinding presented methodological challenges—a single investigation achieved double-blind conditions, whereas remaining studies demonstrated various methodological constraints regarding blinding protocols. Quality evaluation outcomes for the literature are depicted through Figures 2, 3.

**Table 2:** Basic characteristics of included studies.

Author	Type of intervention	Sample size (M/F)	T/C	source	Age (T/C)	Periodicity, frequency, and duration of interventions	Scale used for outcome measurement
Wang et al. (65)	Tai chi	25/27	26/26	China	19.6 ± 1.2	16 weeks, 4 times/week, 60 min/session	CIAS
Ren (66)	Tai chi	24/38	31/31	China	20.1 ± 0.75	8 weeks, 3 times/week, 60 min/session	IAT
Shen (67)	Baduanjin	48/17	31/34	China	19.71 ± 1.71	12 weeks, 3 times/week, 90 min/session	MPAI
Hölzel et al. (68)	Tai chi	24/36	30/30	China	20.1 ± 0.76	8 weeks, 3 times/week, 60 min/session	SAS—SV
Khoury et al. (69)	Mindfulness	22/32	27/27	China	21.3 ± 1.3	8 weeks, 1×/week, 60 min/session	MPIAS
Vago et al. (70)	Dance Sport	6/2	4/4	China	22 ± 2	12 weeks, 3 times/week, 90–120 min/session	SCL—90
Van Dam et al. (71)	Baduanjin	14/530	274/270	China	-	8 weeks, 10 times/week, 20–30 min/times	MPAI
Davidson and Kaszniak (72)	Tai chi	4/48	26/26	China	20.5 ± 1.5	10 weeks, 3 times/week, 60 min/time	MPAI
Baer et al. (73)	Tai chi	31/34	31/34	China	-	10 weeks, 2 times/week, 60 min/session	MPAI
Wilson et al. (74)	Mindfulness	21/38	28/31	China	20.14 ± 1.32	8 weeks, 1/week, 150 min	MPATS
Kirmayer (75)	Qigong	48/17	31/34	China	19.21 ± 1.02	8 weeks, 1/week, 150 min/session	MPATS
Britton et al. (76)	Aerobicdance	41/31	36/36	China	17.45 ± 2.02	12 weeks, 2 times/week, 90 min/session	MPAI
Koch et al. (77)	Mindfulness	-	27/20	China	-	12 weeks, 3 times/week, 30 min/session	SAS-C
Streeter et al. (78)	Baduanjin	48/17	31/34	China	18.95 ± 0.89	8 weeks, 1/week, 150 min/session	MPAI
Heijnen et al. (79)	Mindfulness	13/11	12/12	China	-	12 weeks, 3 times/week, 90 min/session	MPAI
Cross et al. (80)	Mindfulness	-	30/30	China	-	6 weeks, 1/week, 90 min/session	SAS-CA
Zhang et al. (81)	Aerobicdance	0/60	30/30	China	18.83 ± 0.87	8 weeks, 1/week, 120 min/session	MPAI
Li et al. (82)	Aerobicdance	-	8/8	China	20.12 ± 1.54	16 weeks, 2 times/week, 30 min/session	MPAI
Zhang et al. (83)	Baduanjin	26/34	30/30	China	-	6 weeks、3 times/week、90 min/session	MPATS
Zou et al. (84)	Yoga	22/7	15/14	India	22.9 ± 6.5	8 weeks, 3 times/week, 60 min/session	S-IAT
Liu et al. (85)	Mindfulness	43/25	34/34	China	20.36 ± 2.14	8 weeks, 7 times/week, 35 min/session	PVGUA
Wang et al. (86)	Mindfulness	7/30	22/15	China	20.05 ± 1.05	8 weeks, 5 times/week, 60 min/session	MPAI
Luo et al. (87)	Mindfulness	7/12	9/10	China	-	8 weeks, 7 times/week, 90 min/session	MPAI
Maraz et al. (88)	Mindfulness	27/41	34/34	China	18–22	8 weeks, 1/week, 90 min/session	IAT

T, Treatment group; C, Control group; CIAS, Chinese Internet Addiction Scale (Chen); IAT, Internet Addiction Test (Young); MPAI, Mobile Phone Addiction Index (Leung); SAS-SV, Smartphone Addiction Scale-Short Version; SCL-90, Symptom Checklist-90 (Young’s Internet Addiction Related Psychological Scale); MPATS, Mobile Phone Addiction Tendency Scale for College Students (Xiong); SAS-C, The Smartphone Addiction Scale for College Students (Su Shuang); SAS-CA, Adult Smartphone Addiction Scale (Chen Huan); PVGUA, Pathological Online Game Usage Questionnaire.

### Network relationship diagram of included studies

**Figure 4:**
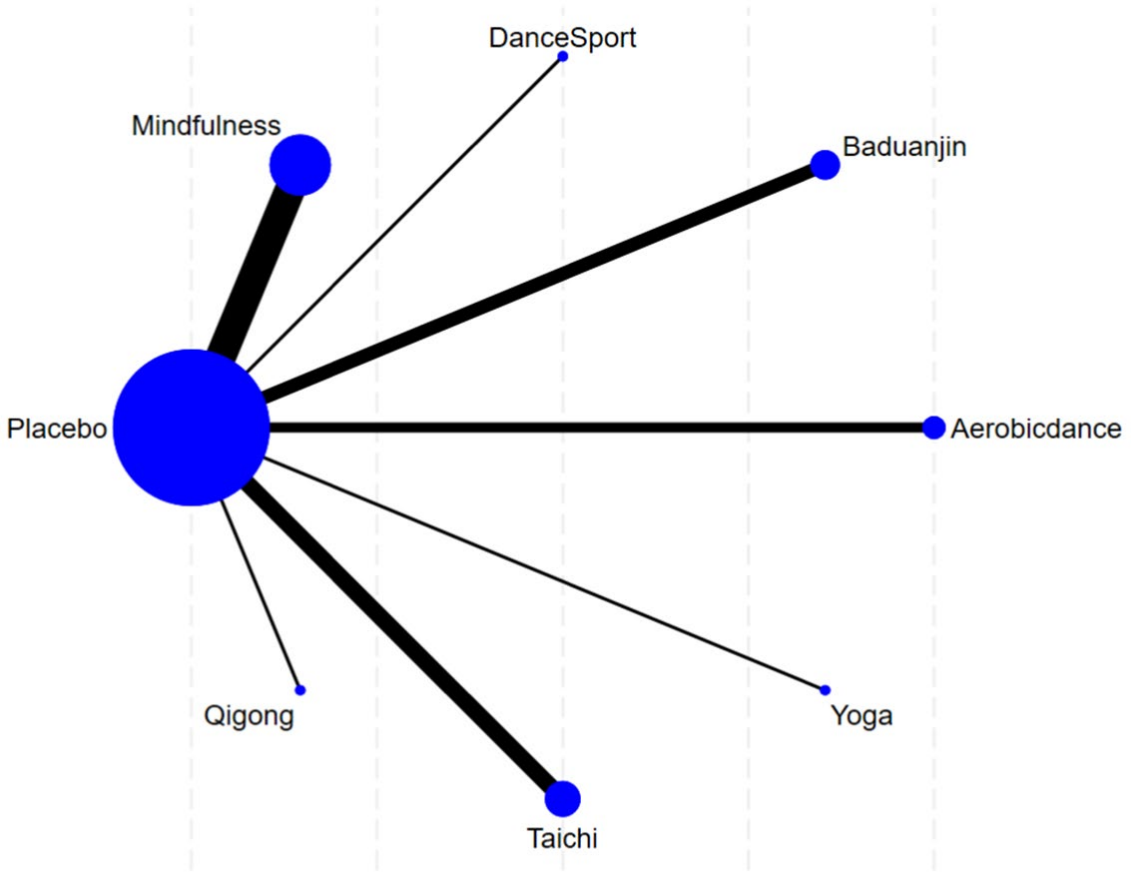
Network evidence graph.

In Figure 4, the seven nodes represent seven intervention measures, with lines between nodes indicating direct comparisons. Within the network diagram, connecting lines vary in width to reflect frequency of direct analytical comparisons made between intervention pairs. The intervention groups include Tai Chi, Mindfulness, Baduanjin, Qigong, Aerobic dance, Yoga, and Dance Sport, while the control group (Placebo) consisted of unstructured physical activity.

### Network meta-analysis

#### Global consistency test

Analysis of consistency revealed several interventions demonstrating superior efficacy in ameliorating IAD symptoms when compared to Placebo (*p* < 0.05). These interventions included: Mindfulness practices showing [*SMD* = −13.33, *95%CI*(−7.42,19.25)]; Taichi demonstrating [*SMD* = −10.91, *95%CI*(−18.71,−3.11)]; Yoga yielding [*SMD* = −11.60, *95%CI*(−28.20,5.00)]; and Qigong with [*SMD* = −7.87, *95% CI*(−24.61,8.87)].

**Figure 5:**
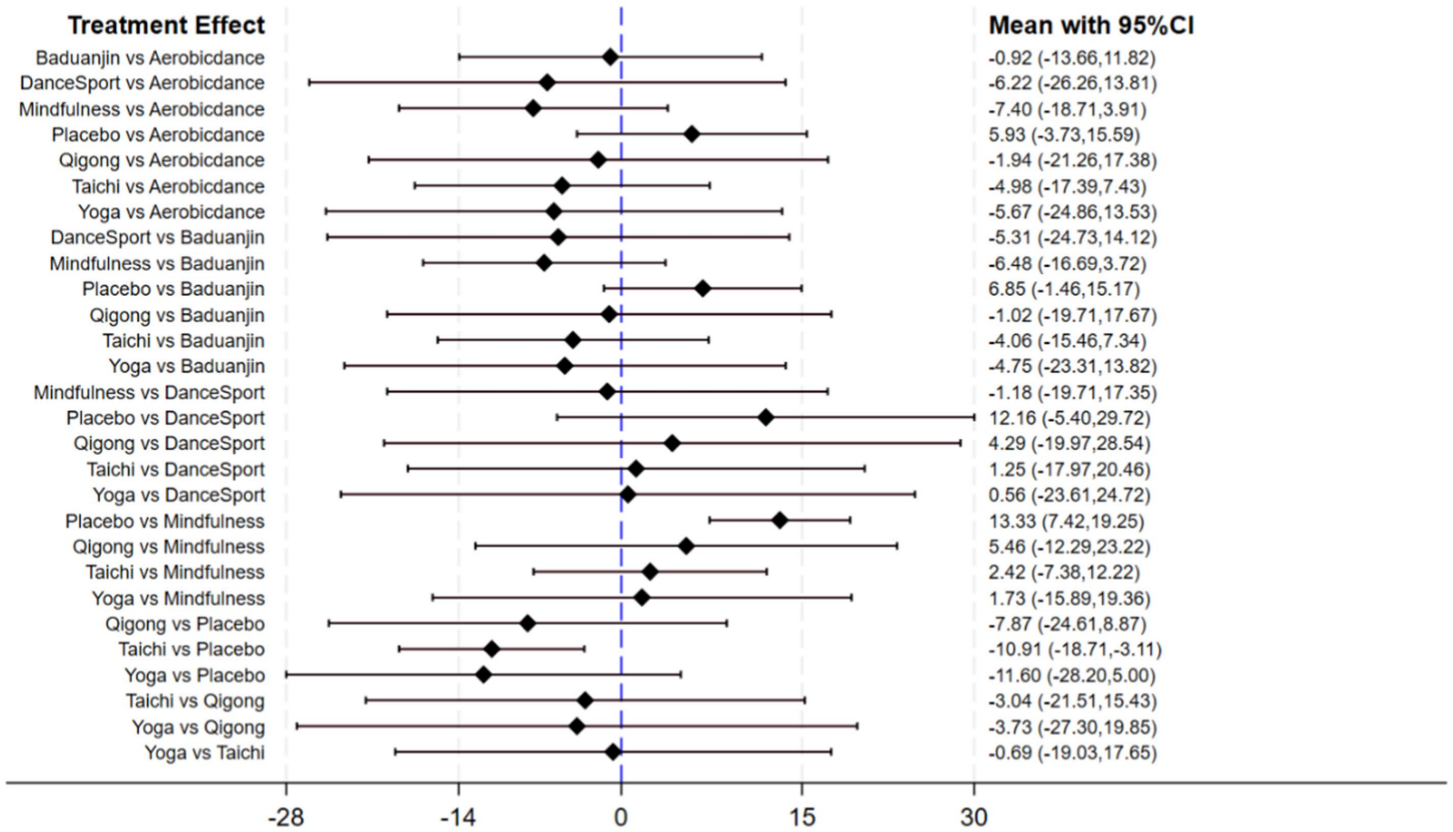
Pairwise comparison forest plot (internet addiction).

Comparative analyses between intervention pairs yielded no statistically meaningful differences among the therapeutic approaches. Specifically, Mindfulness showed comparable effectiveness when evaluated against Taichi, Qigong, and Yoga (*p* > 0.05). Similarly, DanceSport interventions demonstrated equivalent efficacy when compared with Baduanjin and Aerobicdance (*p* > 0.05). Furthermore, no significant variations in therapeutic outcomes emerged from pairwise comparisons across all mind–body exercise modalities, as evidenced by the high *p*-values (ranging from 0.978 to 1.000) shown in Table 3. The *SUCRA* analysis indicated that Mindfulness (76.3%), DanceSport (64%), and Yoga (63.1%) ranked highest in probability of being the most effective interventions for internet addiction symptom improvement, while Placebo ranked lowest (7.4%). Detailed comparative data and visual representations are available in Table 4 and Figure 5, respectively.

**Table 3:** Local inconsistency test.

Side	Direct		Indirect		Difference		
	Coef.	Std. Err.	Coef.	Std. Err.	Coef.	Std. Err.	*P* > |z|
A E	5.936589	4.977863	5.787588	36.73271	0.1490007	37.06844	0.997
B E	6.849274	4.24446	12.11999	194.9034	−5.270713	194.9491	0.978
C E	12.158	8.964714	11.86763	284.5311	0.2903672	284.6749	0.999
D E	13.33513	3.021767	11.82457	106.4293	1.510556	106.4722	0.989
E F	−7.87	8.542452	12.23379	407.2879	4.363792	407.375	0.991
E G	10.91157	3.981224	−11.8777	182.7063	0.9661367	182.75	0.996
E H	−11.6	8.478139	11.46767	215.6887	−0.1323277	215.8551	1.000

A, Aerobicdance; B, Baduanjin; C, DanceSport; D, Mindfulnesst; E, Placebo; F, Qigong; G, Taichi; H, Yoga.

**Table 4:** Cross-comparison results of different mind–body exercise interventions.

Relative effects								
Yoga								
*ES* = −0.69, *95%CI* (−19.03,17.65)	Taichi							
*ES* = 0.56, *95%CI* (−23.61,24.72)	*ES* = 1.25, *95%CI* (−17.97,20.46)	Mindfulness						
ES = −3.73, 95%CI (−27.30,19.85)	*ES* = −3.04, *95%CI* (−21.51,15.43)	*ES* = −4.29, *95%CI* (−28.54,19.97)	Sportdance					
*ES* = −11.60, *95%C*I (−28.20,5.00)	*ES =* −10.91, *95%CI* (−18.71,−3.11)	*ES* = −12.16, *95%CI* (−29.72,5.40)	*ES* = 7.24, *95%CI* (4.56,9.92)	Qigong	*Placebo*			
*ES* = 1.73, *95%CI* (−15.89,19.36)	*ES =* 2.42, *95%CI* (−7.38,12.22)	*ES =* 1.18, *95%CI* (−17.35,19.71)	*ES =* −3.04, *95%CI* (−18.71,−3.11)	ES = −3.04, 95%CI (−18.71,−3.11)	*ES =* −3.04, *95%CI* (−18.71,−3.11)	Mindfulness		
*ES =* −4.75, *95%CI* (−23.31,13.82)	*ES =* −4.06, *95%CI* (−15.46,7.34)	*ES =* −5.31, *95%CI* (−24.73,14.12)	*ES =* −3.04, *95%CI* (−18.71,−3.11)	ES = −3.04, *95%CI* (−18.71,−3.11)	*ES =* −3.04, *95%CI* (−18.71,−3.11)	ES = −6.48 *95%CI* (−16.69,3.72)	Baduanjin	
*ES =* −5.67, *95%CI* (−24.86,13.53)	*ES =* −4.98, *95%CI* (−17.39,7.43)	*ES* = −6.22, *95%CI* (−26.26,13.81)	*ES* = −3.04, *95%CI* (−18.71,−3.11)	ES = −3.04, *95%CI* (−18.71,−3.11)	ES = −1.02, *95%CI* (−19.71,17.67)	ES = −7.40, *95%CI* (−18.71,3.91)	ES = −0.92, *95%CI* (−13.66,11.82)	Aerobicdance

#### Local inconsistency test

**Figure 6:**
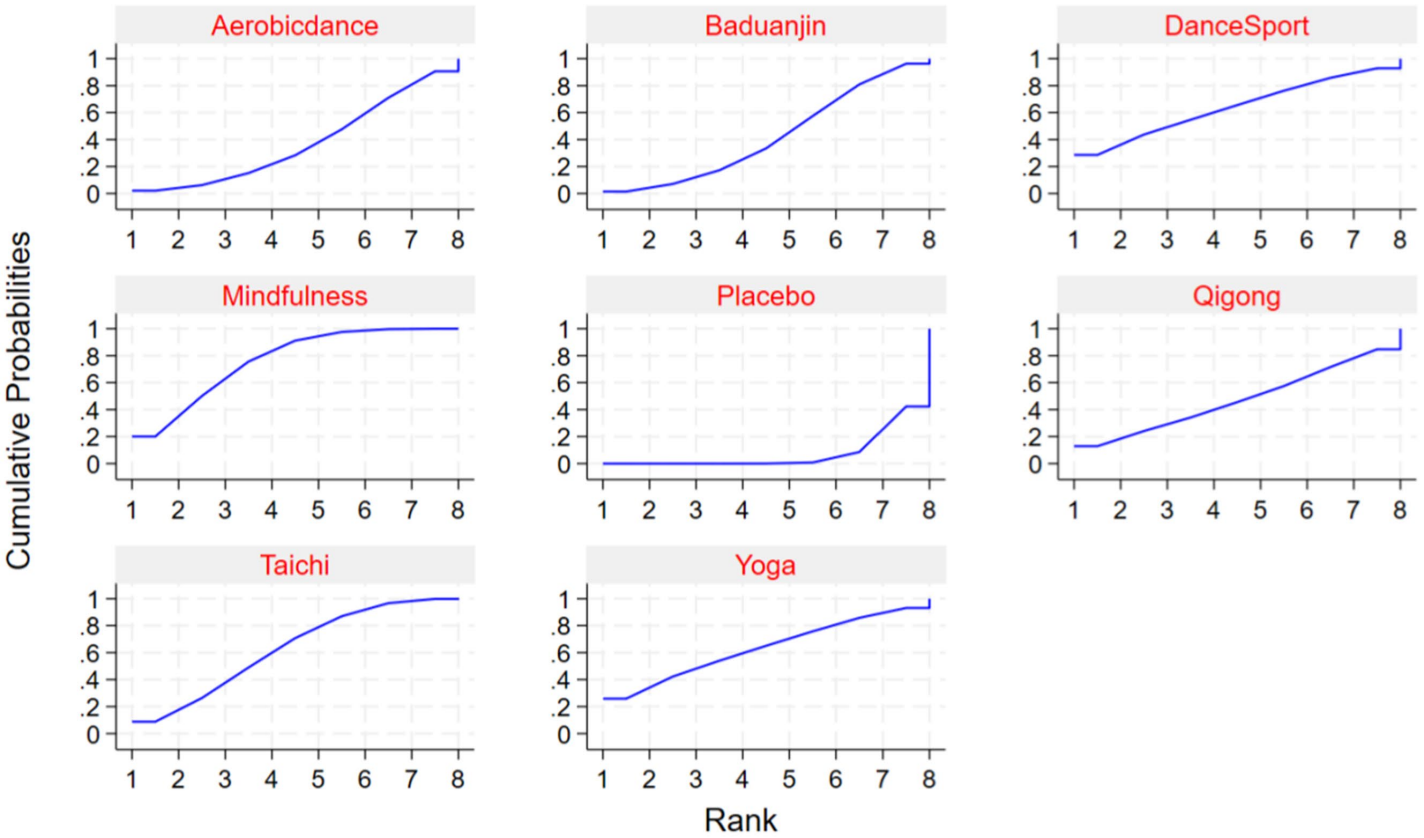
*SUCRA*-based effectiveness analysis of mind–body interventions.

Local inconsistency testing using the node-splitting method showed *p* > 0.05 for comparisons between all mind–body exercise therapies, indicating no statistically significant inconsistency and suggesting good consistency (Figure 6).

### Rank of intervention effectiveness

#### Assessment of publication bias and study size impact

**Figure 7:**
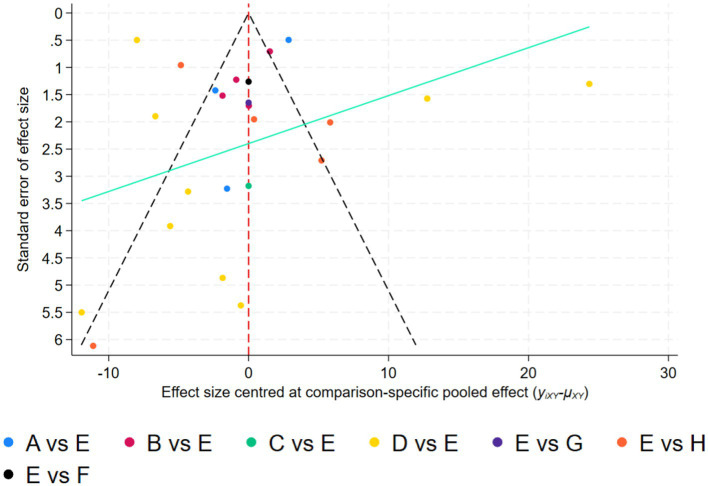
Adjusted comparison funnel plot. A, Aerobicdance; B, Baduanjin; C, DanceSport; D, Mindfulness; E, Placebo; F, Qigong; G, Taichi; H, Yoga.

Analysis of potential reporting bias employed comparison-adjusted funnel plotting methodology. Examination revealed symmetrical distribution patterns relative to the null axis, suggesting minimal influence of study size variation and limited publication bias concerns. Detailed visual representation appears in Figure 7.

## Discussion

Based on our network meta-analysis of mind–body interventions for Internet Addiction Disorder (IAD), Mindfulness demonstrated superior efficacy among the evaluated approaches, with a SUCRA value of 76.3%. DanceSport (SUCRA = 64%), Yoga (SUCRA = 63.1%), and Taichi showed comparable therapeutic benefits. Qigong and Baduanjin exhibited moderate effectiveness, while Aerobicdance (SUCRA = 37.3%) displayed more modest outcomes compared to other mind–body practices. All interventions substantially outperformed placebo conditions, indicating the clinical value of these approaches for IAD management (Table 5).

**Table 5:** Probability rankings and *SUCRA* values for internet addiction symptom improvement across therapeutic modalities.

Norm	Treatment	*SUCRA* (%)	PrBest	MeanRank	Arrange in order
IAD	Mindfulness	76.3	20.1	2.7	1
	DanceSport	64	28.7	3.5	2
	Yoga	63.1	25.9	3.6	3
	Taichi	62.6	8.8	3.6	4
	Qigong	47.2	12.9	4.7	5
	Baduanjin	42	1.5	5.1	6
	Aerobicdance	37.3	2.2	5.4	7
	Placebo	7.4	0	7.5	8

The therapeutic effects of these mind–body interventions can be explained through several complementary neurobiological mechanisms:

Mindfulness’s superior efficacy can be attributed to its multifaceted neurobiological effects. At the neuroplasticity level, Tang et al. (8) found that mindfulness training significantly enhances connectivity between the default mode network and executive control network, optimizing attention regulation and self-awareness capabilities; Yang and Zeng (44) further discovered that 8 weeks of continuous mindfulness training significantly improves gray matter density in the prefrontal cortex and anterior cingulate cortex regions, and these structural changes directly enhance cognitive control ability, contributing to improved executive function in IAD patients. At the neurotransmitter regulation level, Zhang et al. (45) systematic review revealed several neurochemical regulatory pathways of Mindfulness: improving emotional state by stabilizing serotonin levels, alleviating anxiety symptoms by promoting GABA release, while reducing chronic stress response by inhibiting cortisol secretion. In terms of social cognition, Xiao et al. (46) research found that Mindfulness can significantly enhance participants’ self-awareness and interpersonal functioning by activating the insula and strengthening the functional connectivity between the anterior cingulate cortex and prefrontal regions.

However, despite these promising findings, several critical limitations of mindfulness interventions for IAD warrant careful consideration. Zhang et al. (47) comprehensive critical evaluation highlights significant methodological challenges in mindfulness research, including inconsistent operational definitions, inadequate control conditions, and limited measurement precision. These issues potentially confound the interpretation of mindfulness efficacy data in IAD treatment. Lan et al. (48) further argue that the neuroplasticity changes attributed to mindfulness practice often lack specificity and may be influenced by expectancy effects or general relaxation responses rather than mindfulness-specific mechanisms. Regarding clinical implementation, Ren et al. (49) identified substantial heterogeneity in individual responses to mindfulness interventions, with factors such as severity of addiction, comorbid conditions, and pre-existing attentional capacities significantly moderating treatment outcomes. This variability suggests that mindfulness may not be universally effective for all IAD patients. Furthermore, Xie (50) longitudinal study revealed significant attrition rates (averaging 25%–30%) in mindfulness programs for addiction disorders, indicating challenges in treatment adherence that may limit real-world effectiveness. Additionally, the cultural adaptability of traditional mindfulness protocols remains questionable when applied across diverse populations with varying cultural conceptualizations of attention training and mental health, as demonstrated by Kirmayer’s cross-cultural analysis (51). From a neurocognitive perspective, Britton’s research suggests that enhanced awareness through mindfulness might temporarily increase distress in some individuals with addiction disorders by heightening consciousness of withdrawal symptoms before therapeutic benefits emerge (52). These limitations underscore the importance of developing personalized approaches to mindfulness intervention for IAD, rather than adopting a one-size-fits-all approach.

Dance Sport, Yoga, and Aerobic Dance exert therapeutic effects on Internet Addiction Disorder (IAD) through distinct yet complementary neurobiological mechanisms. Dance Sport (SUCRA = 64%) enhances frontal-striatal circuit connectivity through complex sequential movements, with Li (53) demonstrating its ability to upregulate dopamine in reward pathways while simultaneously improving cognitive flexibility. This remodeling of reward circuitry provides IAD patients with alternative pleasure stimuli beyond digital engagement. Yoga (SUCRA = 63.1%), as investigated by Lu et al. (54), operates primarily through autonomic nervous system regulation, increasing parasympathetic activation and GABA levels, which directly counters the sympathetic hyperarousal common in excessive internet use. Its emphasis on breath-body integration strengthens interoceptive awareness, addressing the bodily disconnection often experienced during prolonged screen time. Meanwhile, Aerobic Dance (SUCRA = 37.3%), while showing lower efficacy, contributes through enhanced brain-derived neurotrophic factor (BDNF) production as demonstrated by Yang (55), supporting hippocampal neurogenesis and resilience against stress-induced relapse patterns. The rhythmic, synchronized movements across all three modalities also enhance social synchrony circuits, with Dai (56) finding increased mirror neuron system activation, potentially offering a neural substrate for rebuilding real-world social connections that may have atrophied during internet dependency.

Regarding traditional Chinese health-promoting exercises, our study found that Baduanjin, Tai Chi, and Qigong all showed significant effects in improving IAD. The mechanism of these effects can be elaborated from both modern neuroscience and traditional Chinese medicine perspectives. From a neuroscience perspective, Liu (57) found through systematic review and meta-analysis that mind–body exercises like Tai Chi and Qigong can significantly improve executive function, closely related to prefrontal cortex activation. Improved executive function directly affects individual self-control ability, crucial for IAD rehabilitation. Regarding neurotransmitter regulation, Zhang (58) research confirmed that traditional exercise interventions can significantly improve anxiety and depression symptoms, related to the regulation of neurotransmitters like serotonin. From a traditional Chinese medicine perspective, TCM emphasizes the unity of “form, qi, and spirit, “improving overall health by regulating physical form, breathing, and psychological state. As typical TCM exercise therapies, Tai Chi, Baduanjin, and Qigong emphasize training in breathing regulation, movement coordination, and mental focus (59). Yu et al. (60) systematic review and meta-analysis showed that exercises incorporating meditation elements (like Tai Chi and Qigong) have significant emotional regulation effects. Liao et al. (61) found that Qigong interventions specifically target the dysregulated attentional networks often observed in internet addiction, helping practitioners develop greater attentional flexibility and emotional regulation through its meditative movement practices and energy cultivation techniques.

Our findings both align with and differ from related domestic and international research. For instance, Zhu (62) meta-analysis found that Tai Chi’s effect size on improving mental health [*SMD* = −0.89, *95%CI* (−1.40,−0.38)]was smaller than our results [*SMD* = −10.91, *95%CI* (−18.71,−3.11)]. These differences may arise from several factors. First, Liu et al.’s study included both quasi-experimental designs and RCTs, while our study strictly focused on RCTs, which enhanced the methodological rigor of our findings. Second, Liu et al. used depression scale scores as the primary outcome measure, whereas our study directly assessed IAD symptom severity. Although depression and IAD demonstrate certain comorbidity and symptom overlap, they represent distinct psychopathological dimensions. Third, our study implemented more stringent quality evaluation criteria and utilized net change values for baseline adjustment, which contributed to more precise and reliable results. Similarly, Tadpatrikar et al. (63) systematic review confirmed the positive impact of mind–body exercise. However, the intervention effects in these studies were lower than the effect sizes observed in our research, possibly due to factors such as: (1) differences in intervention duration, with previous studies typically lasting 4–6 weeks while ours continued for 8–16 weeks, allowing more time for mind–body exercise effects to fully manifest; (2) differences in assessment tools, as our study employed a more comprehensive IAD evaluation system. Additionally, our study featured relatively higher intervention frequency and single-session duration, potentially enhancing intervention effects.

Meanwhile, Placebo’s significantly lower ranking stems from its lack of structured neural engagement, absence of specific therapeutic mechanisms, and failure to provide consistent activation of the prefrontal circuits necessary to counter IAD’s neurobiological effects. This pronounced difference between active interventions and placebo confirms the clinical value of structured mind–body approaches for IAD treatment.

This ranking aligns with the “Comprehensive Exercise Intervention Theory” proposed by Wu et al. (64), which suggests that intervention effectiveness positively correlates with the following factors: (1) moderate exercise intensity, (2) complexity of cognitive engagement, (3) richness of social interaction, and (4) diversity of emotional regulation. Dance Sport demonstrates clear advantages in all four dimensions, thus achieving the best results. While Tai Chi and Baduanjin also possess multi-dimensional intervention characteristics, they are relatively weaker in intensity and social aspects. Mindfulness training primarily targets a single dimension (attention and emotional regulation), resulting in lower overall effectiveness.

This study has certain limitations. First, although we strictly included RCT studies and none of the included studies involved clinical patients, dietary intake and daily activities were not strictly controlled between the exercise and control groups during the intervention, which may affect the results. Second, literature searches were limited to Chinese and English, not covering research in other languages, potentially affecting the comprehensiveness of the literature and external validity of the research results. Finally, due to the use of different IAD assessment tools across studies, there exists measurement tool heterogeneity. To address this, we used standardized mean difference to combine effect sizes, ensuring comparability of outcome variables and minimizing the impact of measurement tool differences.

Future research could explore the long-term effects of mind–body exercise therapy on IAD while controlling for diet and daily activities. It is also recommended to extend research to special occupational groups (such as military personnel, firefighters, medical staff, and other high-stress occupational groups) and special physical and mental condition groups (such as minors, disabled persons, chronic disease patients, etc.), exploring differentiated effects and intervention strategies of mind–body exercise therapy across different populations. Additionally, expanding literature search scope to include research in other languages would help improve research comprehensiveness and external validity. It is recommended to conduct higher quality multi-center, large-sample RCT studies focusing on: (1) adopting rigorous experimental designs such as stratified randomization and intention-to-treat analysis; (2) conducting dose–response relationship analysis; (3) setting long-term follow-up observation points, which will provide more reliable evidence support for developing evidence-based, personalized IAD intervention strategies.

Although the literature search of this study covers major international databases, a significant limitation is that all included studies are from Chinese population. This geographical distribution bias may reflect the special contributions of Chinese researchers in the field of mind body intervention research, but it also limits the cross-cultural dissemination of the results. Cultural specificity plays an important role in the study of addictive behavior, especially in Internet use patterns and acceptance of mental health interventions. It is worth noting that Tai Chi and Qigong, as traditional practices originating from China, may benefit from enhanced cultural identity and intrinsic motivation among local populations, resulting in greater intervention effects. These socio-cultural factors may partially explain the significant effect we observed. Future research should expand to populations with different cultural backgrounds, evaluate the applicability of these physical and mental interventions on a global scale, and may require appropriate adjustments for different cultural backgrounds to maximize intervention effectiveness.

## Conclusion

Current evidence suggests that mind–body exercise therapy can effectively improve IAD symptoms in young adults. Among the interventions studied, Mindfulness demonstrated the strongest therapeutic effect (*SUCRA* = 76.3%), followed by DanceSport (*SUCRA* = 64%), Yoga (*SUCRA* = 63.1%), and Taichi (*SUCRA* = 62.6%). Given its superior effectiveness, Mindfulness should be prioritized in IAD intervention programs where feasible. However, all seven interventions showed significant improvements compared to controls, suggesting that selection of specific mind–body exercises can be tailored to individual preferences and practical constraints.

## Data Availability

The raw data supporting the conclusions of this article will be made available by the authors, without undue reservation.
