# Supplementary material for: A network meta-analysis of mind–body exercise interventions for internet addiction symptoms in young adults
Source: Front Public Health. 2025 Jun 18;13:1565372. doi: 10.3389/fpubh.2025.1565372 (PMC12213556; doi:10.3389/fpubh.2025.1565372)
Supplement: Supplementary file 2 [file Table_2.DOCX]

| 序号 | 作者 | 年份 | 实验组 | | 对照组 | | 干预措施  E/C | 干预时间 | 结局指标 | 单盲/  双盲 |
| --- | --- | --- | --- | --- | --- | --- | --- | --- | --- | --- |
|  |  |  | M | W | M | W |  |  |  |  |
| 1 | 杨翠英 | 2017 | 12 | 14 | 13 | 13 | 24简式太极拳/无 | 16周  每周四次（60min） | CIAS  血浆多巴胺、  β-内啡肽 |  |
| 2 | Xueqing Zhang | 2023 | 12 | 19 | 12 | 19 | 杨氏24式太极拳/无/常规运动（篮球等） | 8周  每周三次  （60min） | PSQI  SDS  SAS  FS-14 |  |
| 3 | Tao Xiao | 2021 | 24 | 7 | 24 | 10 | 八段锦/篮球/ | 12周  每周3次  （90min） | MPAI  焦虑、压力、信心、孤独 | 无 |
| 4 | Chunping Lu | 2020 | 24 | 7 | 24 | 10 | ME-Qigong/CBT/无 | 12周  每周2次  （90min） | MAPI*  孤独（SRAS）  焦虑（ULS-8）  压力（PSS-14） | 无 |
| 5 | Ling Yu | 2021 | 0 | 30 | 0 | 30 | 健美操/无 | 16周  每周3次  （60min） | MAPI  焦虑（SAS）  抑郁（SDS） | 无 |
| 6 | 朱淦芳 | 2017 | 13 | 17 | 13 | 17 | 24式太极拳/无 | 8周  每周3次  （60min） | MPAYS |  |
| 7 | Kexin Zhang | 2024 | 12 | 18 | 12 | 18 | 24式太极拳和太极拳八法五部/无 | 8周 | SAS-SV  SDS  SAS  RSES  FS-14 | 无 |
| 8 | YUKUN LAN | 2018 | 27 |  | 27 |  | 团体正念 | 8周  每周1次  （60min） | MPIAS | 无 |
| 9 | 刘诗洁 | 2022 | 30 |  |  |  | 八段锦 | 10周  每周2次  （60min） | MAPI | 无 |
| 10 | 任建华 | 2014 | 3 | 1 | 3 | 1 | 体育舞蹈 | 12周  每周3次  90min-120 | SCL-90 | 无 |
| 11 | 解飞 | 2019 | 162 |  | 152 |  | 健康教育、八段锦/健康教育 | 2个月  每周10次  20-30min | MPAI  SDS  SAS  CAOT | 无 |
| 12 | 张缘 | 2018 | 3 | 23 | 4 | 28 | 太极拳/无 | 10周  每周3次  60min | MPAI | 无 |
|  |  |  |  |  |  |  |  |  |  |  |
| 13 | 杨爱华 | 2019 | 80 |  |  |  | 体育舞蹈 | 15周  每周2次  60min | SAS-C | 无 |
| 14 | 龚宇辉 | 2012 | 16 | 16 |  |  | 二十四式太极拳 | 12周  每周3次  100min | 陈淑惠《中文网络成瘾量表》 | 无 |
| 15 | Xueqing Zhang | 2023 | 12 | 19 |  |  | 二十四式太极拳 | 8周  每周3次  60min | PSQI  SDS  SAS  FS-14 |  |
| 16 | Kexin Zhang | 2024 | 12 | 18 |  |  | 二十四式太极拳 | 8周  每周3次  60min | PSQI  SDS  SAS  FS-14 |  |
| 17 | Fengbo Liu | 2022 | 9 | 13 | 4 | 18 | 正念 | 30min | FMI  MPATS  SCS |  |
| 18 | Nicole D. Anderson | 2007 |  |  |  |  | 正念 | 8周  每周2小时 | PANAS  RSQ |  |

CIAS：《中文网络成瘾量表》强迫症状、退隐症状、耐受症状、人际与健康问题和时间管理问题 5 个维度

PSQI：匹兹堡睡眠质量指数;

SDS：Zung抑郁自评量表;

SAS：Zung焦虑自评量表;

FS-14：疲劳量表-14;

RSES：罗森伯格自尊量表

MAPI*：Xiong, J., Zhou, Z. K., Chen, W., You, Z. Q., Zhai, Z. Y. (2012). Development of the mobile phone addiction tendency scale for college students. Chinese Mental Health Journal, 26, 222–225.

MPIAS：移动网络成瘾量表

智能手机使用
