# Supplementary material for: A network meta-analysis of mind–body exercise interventions for internet addiction symptoms in young adults
Source: Front Public Health. 2025 Jun 18;13:1565372. doi: 10.3389/fpubh.2025.1565372 (PMC12213556; doi:10.3389/fpubh.2025.1565372)
Supplement: Supplementary file 3 [file Table_3.DOCX]

基于四种身心运动疗法对网络成瘾症状干预效果的网状META分析

**摘 要：**背景：网络成瘾(Internet addiction disorder, IAD)是当今严重影响身心健康的复发性精神疾病之一，易导致个体出现神经功能紊乱、免疫低下等生理症状，已成为全球性的公共健康问题。身心运动疗法对网络成瘾的积极影响已被证实，但目前各种身心运动具体疗法对改善IAD的效果不一，尚未发现大规模的临床研究直接比较不同类型的身心运动对网路成瘾患者的疗效。目的：采用网状META分析比较各身心运动疗法改善网络成瘾症状干预的效果。方法：通过检索Web of science、Pubmed、CNKI、VIP等数据库，经过筛选、提取等流程后，使用STATA 18.0进行网状META分析。结果：共纳入10篇文献。同对照组相比，太极拳[SMD=-7.92，95%CI（-11.20，-4.65），P＜0.05]、正念冥想[SMD=-5.30，95%CI（-10.20，-0.41），P＜0.05]、体育舞蹈[SMD=-12.16，95%CI（-19.45，-4.87），P＜0.05]、八段锦[SMD=-7.24，95%CI（-4.56，-9.92），P＜0.05]显著改善网络成瘾症状（P<1.05）。根据SUCRA值，体育舞蹈（SUCRA=95.5）、太极拳（SUCRA=72.8）、正念冥想（SUCRA=50.4）、八段锦（SUCRA=31.2），体育舞蹈排在首位，依次是太极拳、正念冥想、八段锦。结论：体育舞蹈、太极拳、正念冥想、八段锦均能够显著改善网路成瘾症状，太极拳、八段锦均属于气功，极具有推广价值。

**关键词：**身心运动；网络成瘾；网状META

A reticulated META analysis based on the intervention effects of four mind-body exercise therapies on Internet addiction symptoms

**Abstract：**Background: Internet addiction disorder (IAD) is one of the recurrent mental illnesses that seriously affects physical and mental health today, and it is prone to lead to neurological dysfunction, immunocompromise, and other physiological symptoms in individuals, and has become a global public health problem. The positive effects of mind-body exercise therapy on Internet addiction have been demonstrated, but the effectiveness of various mind-body exercise specific therapies in improving IAD is currently variable, and no large-scale clinical studies have been found to directly compare the efficacy of different types of mind-body exercises on Internet addiction patients. OBJECTIVE: To compare the efficacy of various mind-body exercise therapies for improving Internet addiction symptom interventions using reticulated META analysis. METHODS: By searching Web of science, Pubmed, CNKI, VIP and other databases, after screening, extraction and other processes, reticulated META analysis was performed using STATA 18.0. RESULTS: A total of 10 papers were included. Compared with the control group, tai chi [SMD = -7.92, 95% CI (-11.20, -4.65), P < 0.05], positive thinking meditation [SMD = -5.30, 95% CI (-10.20, -0.41), P < 0.05], physical dance [SMD = -12.16, 95% CI (-19.45, -4.87), P < 0.05], and Baduanjin [SMD=-7.24, 95% CI (-4.56, -9.92), P<0.05] significantly improved Internet addiction symptoms (P<1.05). According to SUCRA values, physical dance (SUCRA=95.5), taijiquan (SUCRA=72.8), positive thinking meditation (SUCRA=50.4), and eight-duanjin (SUCRA=31.2), physical dance ranked first, followed by taijiquan, positive thinking meditation, and eight-duanjin. Conclusion: Physical dance, taijiquan, positive thinking meditation, and Baduanjin were able to significantly improve the symptoms of Internet addiction, and taijiquan and Baduanjin belong to qigong, which is extremely valuable for promotion.

**Key words：**Mind-body movement; Internet addiction; Net META

**前言**

随着信息技术的快速崛起，网络已成为民众日常生活不可或缺的一部分。然而过度依赖智能手机等技术可能会患上网络成瘾综合征（Internet addiction disorder, IAD），带来健康风险。IAD是指网络成瘾指由于各种因素导致个体无节制、强迫性、难以摆脱地沉迷于网络，从而造成生理、心理及社会功能受损的成瘾行为^[1]^。网络成瘾不仅会导致个体产生神经功能紊乱、免疫低下等生理症状^[2]^，还会引起认知能力下降、焦虑、抑郁等心理问题^[3-4]^。Olson等人^[5]^的研究中显示，网络成瘾正在逐年递增，已成为全球性的公共健康问题^[6]^。

现阶段IDA的干预手段涉及范围较广，例如药物治疗^[7]^、针灸^[8]^、运动行为^[9]^等，由于药物干预的副作用、联合用药禁忌等问题，非药物干预越来越受到支持^[10]^。体育运动在心理健康中具有的特殊作用，已逐渐被认可^[11-12]^。身心运动（Mind-Body Exercise，MBE）被美国国立卫生研究院、、国家补充和综合健康中心定义为替代、补充药物的一个类别^[13]^。其作为一种多模式的运动，兼具有氧运动、阻力运动等运动方式的综合特征^[14-15]^，其典型特征是缓慢的身体运动，全身的舒展和放松，呼吸的控制和精神意念的集中等结构化运动形式，以中国传统体育太极、健身气功（八段锦）和古印度正念冥想为代表^[16]^，是生理和心理疗法的结合^[17-18]^。与其他有氧或阻力运动相比^[19-20]^，身心运动的优点是节律慢、强度稳定，有利于长期健康发展，并且身心运动不必要使用运动器材设备^[21-22]^，学习成本低，安全性高，这也是它能大规模、多人群推广的缘由。

先前研究认为太极拳、体育舞蹈等身心疗法能够有效改善网络成瘾症状。但目前大多数研究仅涉及单项疗效分析，鲜有同时比较多种身心运动在改善网路成瘾症状效果的循证研究。Neha Pirwani^[23]^等的综述提出，当前研究大部分针对特殊人群，缺乏对多年龄段人群的关注。与仅可直接比较的常规META分析相比，网状META分析通过直接比较或间接比较可以对干预措施的效果进行排序^[24]^。因此，本研究纳入身心运动疗法治疗网络成瘾症状的RCTs，运用网状META分析整合直接与间接比较关系的证据，检验不同身心运动疗法对网络成瘾症状的影响，并比较各疗法功效以及根据疗效进行排序，以期为改善网络成瘾症状提供干预策略的依据和循证证据支持。

**1 资料来源和方法**

本研究申请并已通过PROSPERO平台的注册，注册ID为CRD42022314522。

1.1文献检索

本研究严格按照PRIMSA^[25]^条目规定的流程，并根据循证医学PICOS原则，严格制定文献的检索、纳入、筛选以及排除标准。在Elsevier Sciencedirect、Web of science、Pubmed、Proquest、Scopus、The Cochroae Library、CNKI、WFDSP、VIP数据库以（“身心练习”或“身心运动”或“瑜伽”或“冥想”或“太极拳”或“八段锦”或“气功”或“易筋经”或“五禽戏”）和（“网络成瘾”或“互联网成瘾”或“网瘾”或“社交网络依赖”或“网络依赖”或“数字成瘾”或“网络沉迷”或“成瘾”）和（“随机对照试验”或“对照临床试验”）为中文主题词进行布尔逻辑检索；以("Internet Addiction Disorder"or"Addiction Disorder, Internet"OR ("Addiction Disorders, Internet"or"Disorder, Internet Addiction"or"Disorders, Internet Addiction"or"Internet Addiction Disorders"or"Internet Addiction"or"Addiction, Internet"or "Addictions, Internet"or"Internet Addictions"or"Internet Gaming Disorder"or"Disorder, Internet Gaming"or"Disorders, Internet Gaming"or"Gaming Disorder, Internet"or"Gaming Disorders, Internet"or"Internet Gaming Disorders"or"Smartphone Addiction"or"Addiction, Smartphone"or"Addictions, Smartphone"or"Smartphone Addictions"or"Social Media Addiction"or"Addiction, Social Media"or"Addictions, Social Media"or"Media Addiction, Social"or"Media Addictions, Social"or"Social Media Addictions")and("Mind and body exercise"or"Yoga"or"Meditation"or"Taichi"or"Eightbrocade"or"Qigong"or"Yijinjing"or"Wuqinxi")and("Psychology"or"Depression"or"Anxiety"or"Negative emotion"or"Negative psychology")and("Randomized controlled trial"or "Controlled clinical trial" or"Randomized")为英文主题词进行布尔逻辑检索。语言仅限中文与英文，中文仅收纳核心期刊，检索时间范围从2000年1月截止至2022年4月1日。同时通过追溯相关研究的参考文献等其他途径进行文献获取。以 PubMed 为例，见框1。

框1 PubMed 检索策略

Box1 PubMed search strategy

| #1 ("Mind"[Title/Abstract] OR "body exercise"[Title/Abstract] OR "Mind-body exercise"[Title/Abstract] OR "Taichi"[Title/Abstract] OR "Baduanjin"[Title/Abstract] OR "Mindfulness"[Title/Abstract] OR "Sport Dance"[Title/Abstract]) AND "Internet Addiction Disorder"[MeSH Terms]  #2 "Sports"[Title/Abstract] OR "Sport"[Title/Abstract] OR "Athletics"[Title/Abstract] OR "Athletic"[Title/Abstract]  #3 #1 OR #2  #4 "Internet Addiction Disorder"[Title/Abstract] OR "Addiction Disorder, Internet"[Title/Abstract] OR "Addiction Disorders, Internet"[Title/Abstract] OR "Disorder, Internet Addiction"[Title/Abstract] OR "Disorders, Internet Addiction"[Title/Abstract] OR "Internet Addiction Disorders"[Title/Abstract] OR "Internet Addiction"[Title/Abstract] OR "Addiction, Internet"[Title/Abstract] OR "Addictions, Internet"[Title/Abstract] OR "Internet Addictions"[Title/Abstract] OR "Internet Gaming Disorder"[Title/Abstract] OR "Disorder, Internet Gaming"[Title/Abstract] OR "Disorders, Internet Gaming"[Title/Abstract] OR "Gaming Disorder, Internet"[Title/Abstract] OR "Gaming Disorders, Internet"[Title/Abstract] OR "Internet Gaming Disorders[Title/Abstract] OR Smartphone Addiction[Title/Abstract] OR Addiction, Smartphone[Title/Abstract] OR Addictions, Smartphone[Title/Abstract] OR Smartphone Addictions[Title/Abstract] OR Social Media Addiction[Title/Abstract] OR Addiction, Social Media[Title/Abstract] OR Addictions, Social Media[Title/Abstract] OR Media Addiction, Social[Title/Abstract] OR Media Addictions, Social[Title/Abstract] OR Social Media Addictions[Title/Abstract]  #5 "randomized controlled trial"[Publication Type] OR "controlled clinical trial"[Publication Type]  #6 #3 AND #4 AND #5 AND #6 |
| --- |

1.2 文献纳入标准

本研究仅纳入随机对照试验（Randomized Controlled Trial，RCT）；接受身心运动干预负面情绪RCT实验的大学生（包括轻度焦虑、抑郁倾向或症状），干预组干预措施为含太极拳、正念冥想、八段锦、体育舞蹈中任意一种身心运动形式，对照组干预措施为无规律性体育活动；结局指标为网络成瘾量表（实验组与对照组在干预前后均采用不限类型的网络成瘾量表对大学生进行心理健康测试，干预后与干预前测的值之差小于0，则干预为有效）。

1.3 排除标准

非RCT实验；动物实验研究；描述性与调查性文献、综述类文献、二次研究、学术会议收录文献以及重复发表的文献；实验结果数据未以(x±s)形式呈现或数据无法提取指标的文献；有干预过程中有其他干预措施；数据描述不全或者无法转换。

1.4文献筛选与资料提取

由2名研究人员按照研究策略将所收集到的文献均导于NoetExpress V4.X软件，排除其中重复文献，然后根据标题和摘要分别进行独立筛选。再根据纳入与排除标准，详细阅读全文再次筛选。此后，2人对各自筛选结果进行交叉核对，核对一致则纳入研究，若核对有异由第3名研究人员进入协商，讨论达成一致后共同决定；最终对最终纳入的文献进行信息提取，采用预先制定的信息提取表对纳入研究的文献数据以及文献偏倚风险记录进行归纳。数据提取内容主要包括：（1）纳入文献的基本信息（题目、一作、年份等）；（2）被试特征及信息（样本量、年龄、性别等）；（3）干预及对照详情（类型、持续时间、频次等）；（4）文献质量；（5）结局指标和主要研究结果。

1.5纳入文献偏倚风险评价

采用RevMan5.4版系统评估手册中偏倚风险评估工具，由两名研究人员独立进行纳入研究的质量评价。若评估结果不一致，与另一名研究人员讨论解决。

1.6统计处理

通过Stata17.0软件对数据进行META分析，结局指标为连续性变量。由于纳入的文献中对同一指标采用工具不一，采用标准化均数差（SMD）表示，基线统一调整为α=0.05。采用Q检验和$I^{2}$检验进行异质性检验，发表偏倚分析采用Egger检验方法。当网状Meata分析时，若网状证据图中存在闭环结构，采用节点分析进行不一致性检验。环不一致检验的结果若P>0.05，则进行一致性模型分析。同时使用节点劈裂法进行局部不一致性检验，如若有结果P＜0.05，则遵循该比较的常规META分析中直接比较结果。通过累积排序概率图下面积SUCRA值(surface under the cumulative ranking,SUCRA)进行各干预措施优劣排序（0≤SUCRA≤1）。根据两个结局指标的SUCRA值，采用聚类分层分析探索身心运动疗法这种最优组；发表偏倚采用校正比较漏斗图进行评价。

**2 Meta-analysis results**

2.1 文献检索与筛选情况

初筛获得文献93篇，经过层层筛选，最终纳入10项研究，筛选过程及结果详见图1。


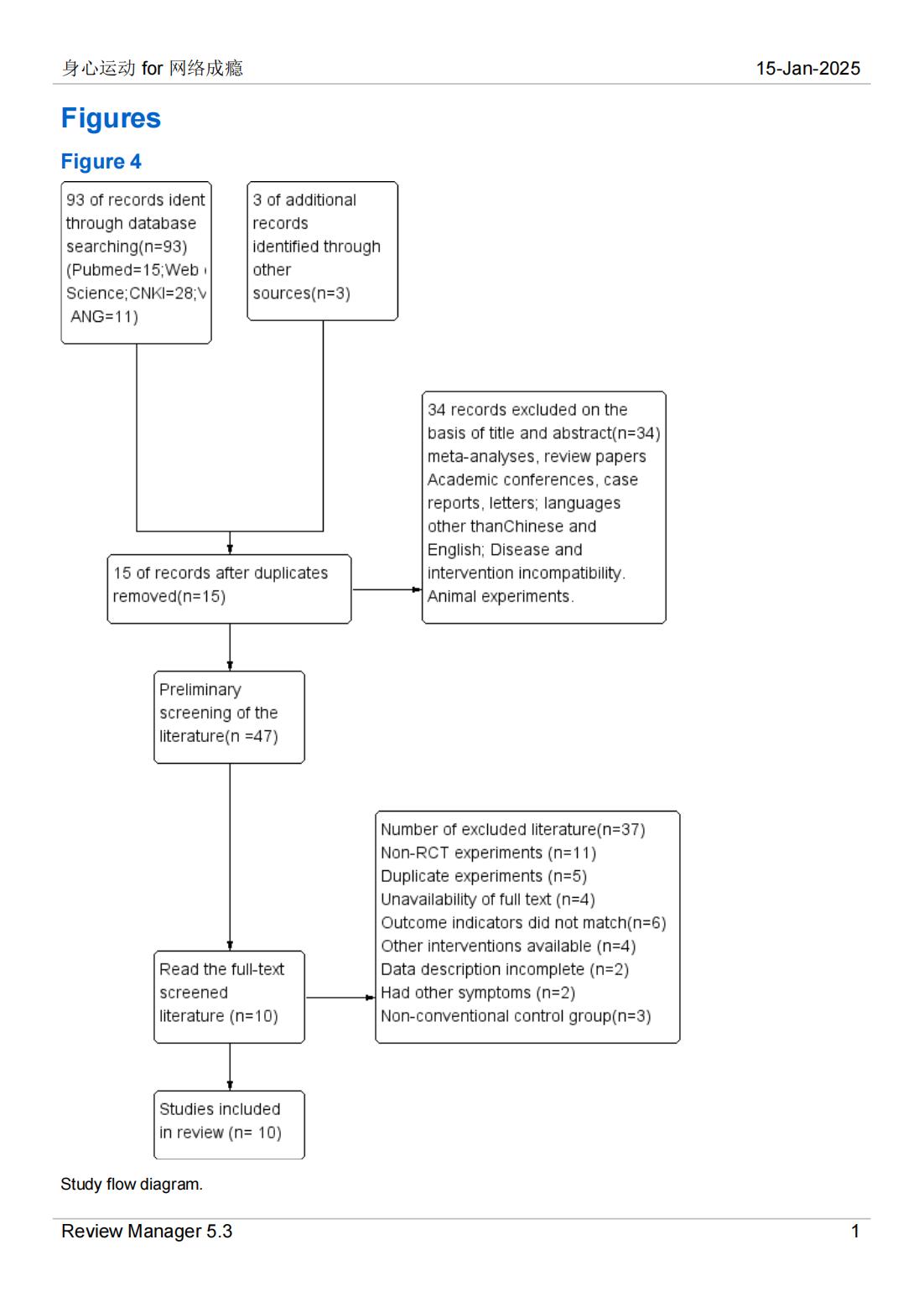


图1 文献筛选流程图

Figure 1 Literature screening flowchart

2.2 纳入研究的文献基本特征及方法学质量评估

最终共纳入11项研究，纳入的全部研究基本特征详见表1，共1306名受试者。另外，由于该运动疗法的特殊性，且进行需签订知情同意书，仅有1篇[34]文章采用双盲，其他研究在盲法实施方面都存在不同程度的局限或问题，文献质量评价结果详见表2、图2。

表1 纳入研究的基本特征

Table1 Basic features of the included studies

| 作者及年份 | 干预  形式 | 样本量  (男/女) | T/C | 来源 | 年龄（T/C） | 干预周期、频率与时间 | 结局指标 | 测量工具 |
| --- | --- | --- | --- | --- | --- | --- | --- | --- |
| 杨翠英2017^[26]^ | 太极拳 | 25/27 | 26/26 | 中国 | 19.6±1.2 | 16周、4次/周、60min/次 | 网络成瘾 | CIAS |
| Xueqing Zhang2023^[27]^ | 太极拳 | 24/38 | 31/31 | 中国 | 20.1±0.75 | 8周、3次/周、60min/次 | 网络成瘾 | IAT |
| Tao Xiao 2021^[28]^ | 八段锦 | 48/17 | 31/34 | 中国 | 19.71±1.71 | 12周、3次/周、90min/次 | 网络成瘾 | MPAI |
| Kexin Zhang 2024^[29]^ | 太极拳 | 24/36 | 30/30 | 中国 | 20.1±0.76 | 8周、3次/周、60min/次 | 网络成瘾 | SAS - SV |
| Yukun Lan 2018^[30]^ | 正念冥想 | 22/32 | 27/27 | 中国 | 21.3 ± 1.3 | 8周、1次/周、60min/次 | 网络成瘾 | MPIAS |
| 任建华2014^[31]^ | 体育舞蹈 | 6/2 | 4/4 | 中国 | 22±2 | 12周、3次/周、90-120min/次 | 网络成瘾 | SCL - 90 |
| 解飞2019^[32]^ | 八段锦 | 14/530 | 274/270 | 中国 | － | 8周、10次/周、20-30min/次 | 网络成瘾 | MPAI |
| 张缘2018^[33]^ | 太极拳 | 4/48 | 26/26 | 中国 | 20.5±1.5 | 10周、3次/周、60min/次 | 网络成瘾 | MPAI |
| 刘诗洁2022^[34]^ | 太极拳 | 31/34 | 31/34 | 中国 | － | 10周、2次/周、60min/次 | 网络成瘾 | MPAI |
| 李毅2022^[35]^ | 正念冥想 | 21/38 | 28/31 | 中国 | ＞18 | 8周、1次/周、150min | 网络成瘾 | MPATS |

注：T：实验组；C：对照组；CIAS：《中文网络成瘾量表》陈淑惠；IAT：网络成瘾测验Young；MPAI：《手机成瘾指数量表》梁永炽；SAS - SV：《智能手机成瘾量表-短版》；SCL - 90：网络成瘾相关心理量表Young；MPATS：《大学生手机成瘾倾向量表》熊婕

表2 纳入研究的文献质量风险偏倚评价结果

Table2 Evaluation results of literature quality risk bias of included studies


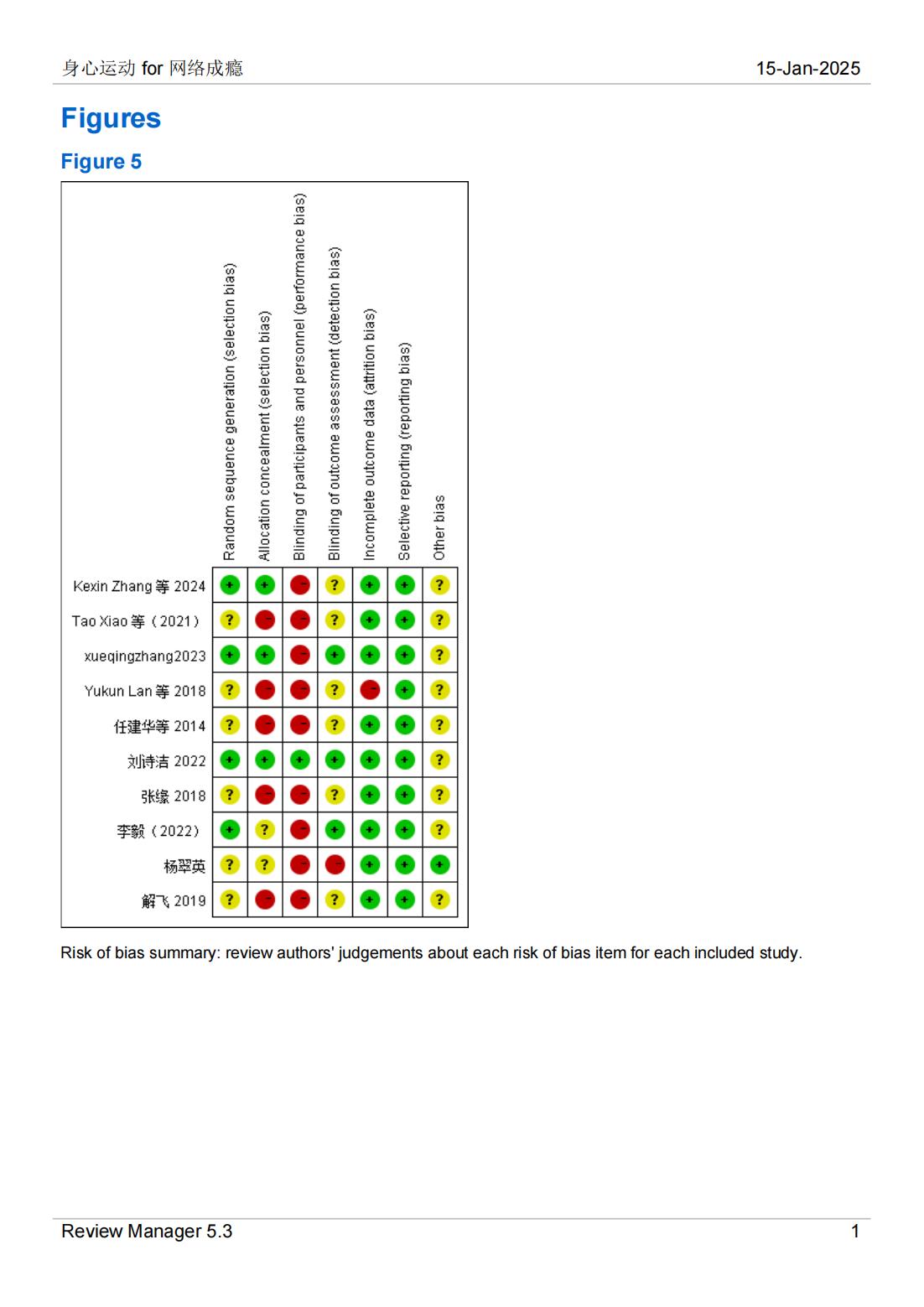


Risk of bias summary: review authors' judgements about each risk of bias item for each included study.


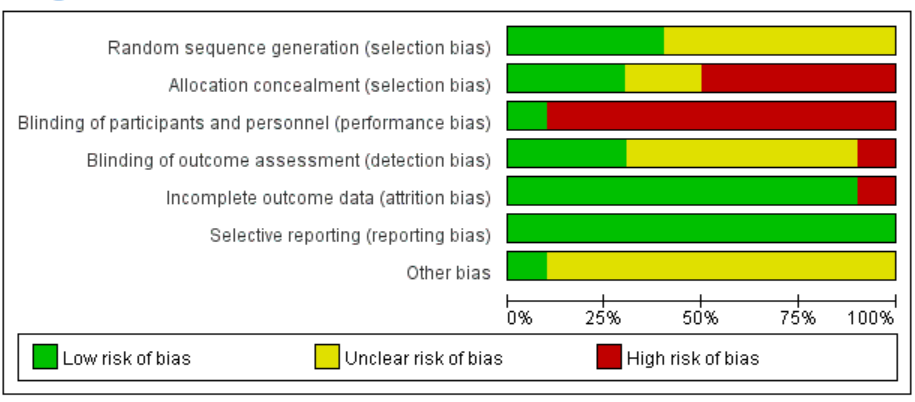


Risk of bias graph: review authors' judgements about each risk of bias item presented as percentages across all included studies.

图2 纳入研究的文献质量风险偏倚评价结果条形图

Fig. 2 Bar graph of the results of quality risk of bias evaluation of the literature of the included studies


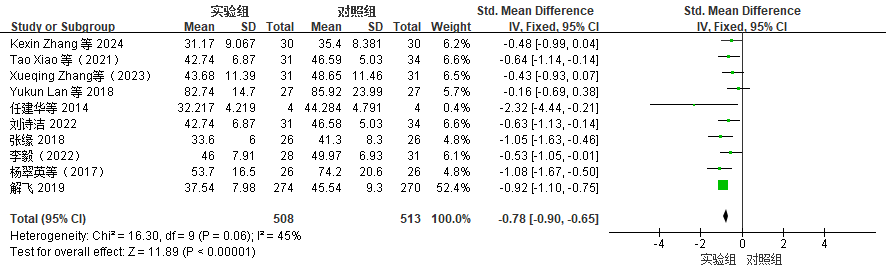


图3 身心运动对网络成瘾的影响

Figure 3 Effect of physical and mental exercise on internet addiction

本研究采用森林图直观展示了纳入荟萃分析的10项研究的效应量估计值及其95%可信区间，见图3。每个研究用方块表示其效应量估计值，方块大小与研究权重成正比，横线代表效应量的可信区间。图底部给出了基于固定效应模型的整体效应量估计值。异质性检验结果为I²=45%，P=0.06，表明各研究间异质性较低，因此采用固定效应模型进行汇总分析是合适的。荟萃分析结果显示，身心运动干预组在改善网络成瘾者心理健康水平方面优于对照组，标准化均数差[SMD=-0.78，95%CI(-0.90,-0.65)]，差异具有统计学意义。该结果为身心运动干预网络成瘾的有效性提供了证据支持。


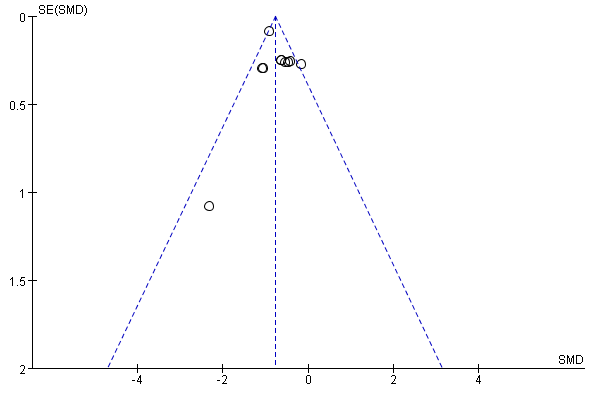


图4 网络成瘾漏斗图

Figure 4 Internet addiction funnel diagram

漏斗图可用于直观评估荟萃分析是否存在发表偏倚。如图4所示，漏斗图以效应量为横轴，以研究精度（标准误的倒数）为纵轴。在没有发表偏倚的理想情况下，研究应围绕真实效应呈现出一个倒漏斗状对称分布。本研究纳入的各项研究点基本围绕总体效应量SMD=-0.78对称分布，未见明显偏离漏斗形状，提示发表偏倚风险较低。大多数研究点位于图的上方，表明纳入研究的整体精度较高，META分析结果可靠。

2.3 纳入研究的网状关系图

图2中5个圆点代表5种干预措施，圆点间的直线代表干预间存在直接比较，直线的粗细代表该两种干预措施直接比较数目的多少。观察组干预包括太极拳、正念冥想、八段锦、体育舞蹈；对照组为无规律性体育活动，见图5。


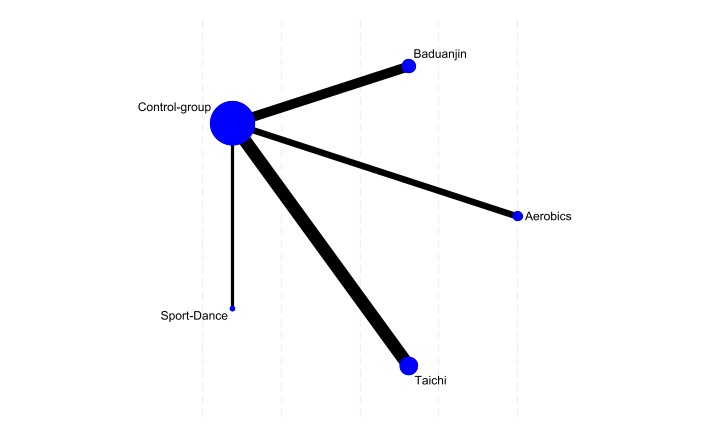


图5 网状证据图

Figure 5 Reticulated evidence map

2.4 网状Meta分析

2.4.1 整体一致性检验

一致性分析结果显示：太极拳[SMD=-7.92，95%CI（-11.20，-4.65），P＜0.05]、正念冥想[SMD=-5.30，95%CI（-10.20，-0.41），P＜0.05]、体育舞蹈[SMD=-12.16，95%CI（-19.45，-4.87），P＜0.05]、八段锦[SMD=-7.24，95%CI（-4.56，-9.92），P＜0.05]均对大学生网络成瘾的改善效果优于无规律性体育活动，且差异均具有统计学意义（P＜0.05）。两两之间的间接比较结果显示，太极拳与正念冥想、体育舞蹈、八段锦的差异无统计学意义（P＞0.05）；正念冥想与体育舞蹈、八段锦的差异亦无统计学意义（P＞0.05）。所有身心运动疗法之间的两两比较结果表明，各疗法在效果上的差异未能达到统计学显著性（P＞0.05）。见图6、表3。

2.4.2 局部不一致检验

通过节点劈裂法对两个指标的分析进行局部不一致性检验，结果显示：所有身心运动疗法之间的比较均是P＞0.05，不一致性均没有统计学意义，说明一致性较好。

2.5 各干预有效性排序

身心运动对网络成瘾症状疗效优劣排序依次为：体育舞蹈（SUCRA=95.5）、太极（SUCRA=72.8）、正念冥想（SUCRA=50.4）、八段锦（SUCRA=31.2）且均优于对照组无规律体育活动（SUCRA=0.1），详见图7、表4。


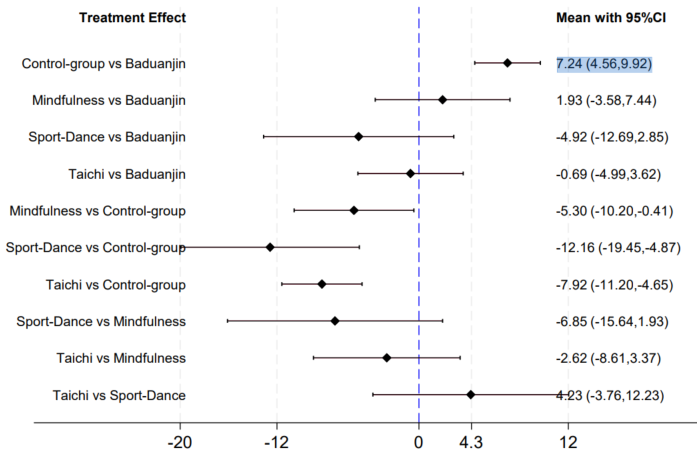


图6 两两对比森林图（网络成瘾）

Figure 6 Two-by-two comparison forest plot (internet addiction)

表3 各身心运动疗法之间效果的交叉比较结果

Table 3 Cross-comparison results of the effects of various psychosomatic motor therapies

| **Relative effects** |  |  |  |  |
| --- | --- | --- | --- | --- |
| Taichi |  |  |  |  |
| ES=4.23  95%CI(-3.76,12.23) | Sport-Dance |  |  |  |
| ES=-2.62 95%CI(-8.61,3.37) | ES=-6.85 95%CI(-15.64,1.93) | Mindfulness |  |  |
| ES=-7.92 95%CI(-11.20,-4.65） | ES=-12.16 95%CI(-19.45,-4.87) | ES=-5.30 95%CI(-10.20,-0.41) | Control-group |  |
| ES=-0.69  95%CI(-4.99,3.62) | ES=-4.92 95%CI(-12.69,2.85) | ES=1.93  95%CI(-3.58,7.44) | ES=7.24  95%CI(4.56,9.92) | Baduanjin |


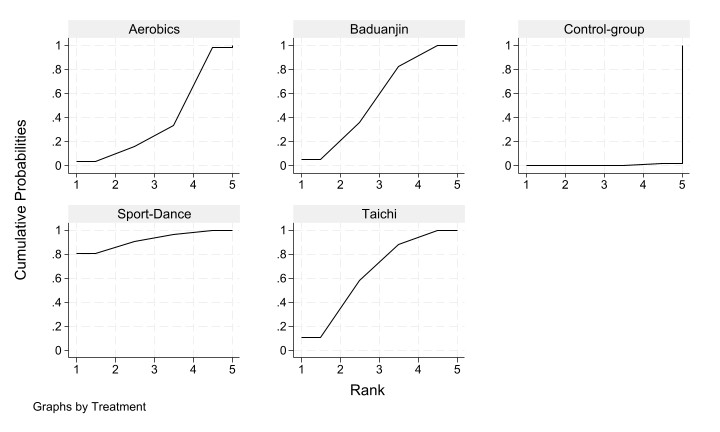


图7 干预效果排序图

Figure 7 Ranking chart of intervention effect

表4 各不同身心运动疗法改善大学生负性心理症状等级概率排序

Table 4 Probability ranking of different psychosomatic motor therapy to improve negative psychological symptoms of college students

| 指标 | 干预措施 | SUCRA  （%） | PrBest | MeanRank | 排序 |
| --- | --- | --- | --- | --- | --- |
| 网络成瘾 | Sport-Dance | 91.1 | 80.9 | 1.3 | 1 |
|  | Taichi | 64.2 | 10.8 | 2.4 | 2 |
|  | Baduanjin | 55.8 | 5.3 | 2.8 | 3 |
|  | Aerobics | 37.6 | 3.0 | 3.5 | 4 |
|  | Control-group | 0.5 | 0.0 | 5.0 | 5 |

2.6 小样本效应或发表偏倚检验

对纳入网状META的研究通过矫正－比较漏斗图进行小样本效估计及发表偏倚检测，纳入研究基本对称，提示当前研究存在小样本效应可能性较小，未发现明显发表偏倚，详见图8。


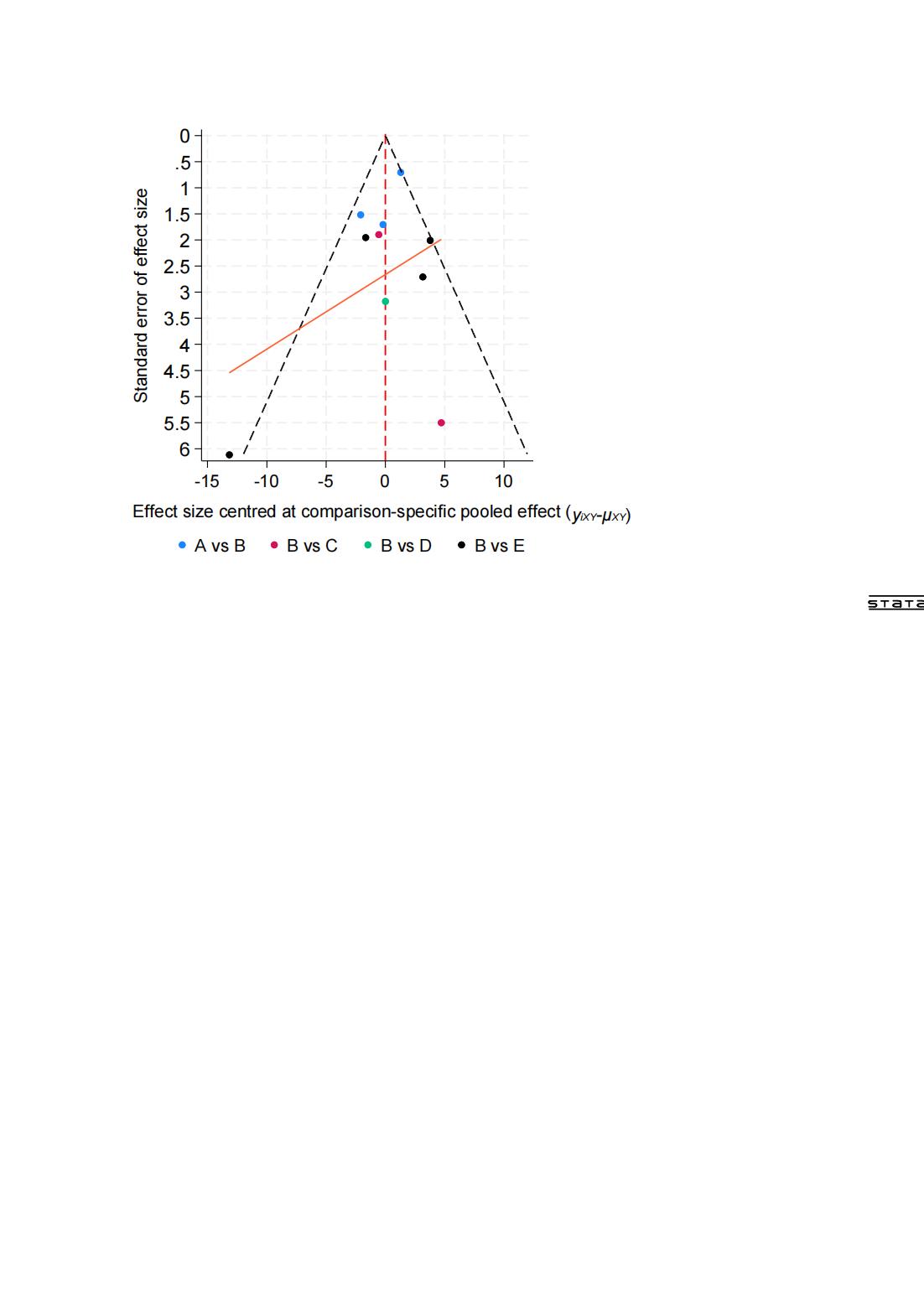


图8 校正比较漏斗图

Figure 8 Correction comparison funnel plot

**3 讨论**

本研究将四种身心运动疗法作为观察组，以无规律性体育活动作为对照组，并仅纳入随机对照试验（RCT）研究。我们采223用RevMan5.4版系统评估手册对纳入RCT研究的偏倚风险进行了评估，并使用网状META分析方法，对各身心运动疗法的效果进行了直接与间接比较，旨在探讨改善大学生网络成瘾的最佳身心运动疗法。本研究的主要发现是：整体来看，身心运动疗法的直接比较和间接比较结果一致，所有干预方式均能显著改善大学生的网络成瘾状况。根据SUCRA排序结果，体育舞蹈（SMD=-12.16，95%CI：-19.45，-4.87，P＜0.05）、太极拳（SMD=-7.92，95%CI：-11.20，-4.65，P＜0.05）、八段锦（SMD=-7.24，95%CI：-4.56，-9.92，P＜0.05）和正念冥想（SMD=-5.30，95%CI：-10.20，-0.41，P＜0.05）同样具有统计学意义，在改善网络成瘾方面表现出了不同程度的效果，尤其是体育舞蹈效果显著优于其他干预方法。

本研究的结果与国内外相关研究既有一致性也存在差异。例如，Liu等^[36]^的META分析研究发现太极拳对改善心理健康的效应量[SMD=-0.89，95%CI（-1.40，-0.38）]小于本研究结果[SMD=-7.92，95%CI（-11.20， -4.65）]。这种差异可能源于以下几个方面：首先，Liu等的研究同时纳入了准实验设计和随机对照试验，而本研究严格限定仅纳入随机对照试验；其次，其研究对象包含了一般人群，而本研究则专注于网络成瘾的大学生群体；最后，本研究采用了更为严格的质量评价标准，并使用了差值比较的净变化量方法进行基线调整，这些方法学上的改进使得本研究的结果更加精确。

同时，Luo等人^[37]^的系统评价也证实了身心锻炼对网络成瘾的积极影响。然而，这些研究中的干预效果都低于我们研究中观察到的效应大小，这可能与以下因素有关：首先，干预期的差异，以前的研究通常持续 4-6 周，而我们的研究持续 8-16 周，让身心锻炼的效果有更多时间充分显现。其次，评估工具的差异，因为我们的研究采用了更全面的网络成瘾评估系统。此外，我们的研究具有相对较高的干预频率和单次会话持续时间，这可能增强了干预效果。

在中医传统健身项目方面，本研究发现八段锦和太极拳对改善大学生的网络成瘾具有显著效果。根据中医“形、气、神”一体化理论，身心一元论强调通过调节形体、呼吸和心理状态来改善个体的整体健康。太极拳和八段锦作为典型的中医运动疗法，注重呼吸调节、动作协调和意念专注的训练，符合中医理论中的“调形、调息、调神”，因此其干预效果非常突出。尤其在本研究中，太极拳的效果次于体育舞蹈，表明中医传统运动疗法在改善大学生的网络成瘾方面也具有强大潜力。

瑜伽和正念冥想虽然在本研究中表现较为温和，但仍显示出一定的效果。瑜伽以其强调身体柔韧性、静心训练、呼吸控制和意识集中相结合的特点，已被广泛证明能改善心理健康，特别是在情绪调节和压力缓解方面。正念冥想通过自我觉察、呼吸调节和注意力集中，有助于减少情绪困扰和增强个体对情境的应对能力，进而缓解网络成瘾相关症状。尽管这些干预方法的效果略逊于运动型身心疗法，但它们依然为缓解网络成瘾提供了有效的辅助手段。

本研究也存在一定的局限性。首先，尽管我们严格纳入了RCT研究，且所有纳入的研究均未涉及临床病患者，但在干预过程中，运动组与对照组的饮食摄入和日常活动并未进行严格控制，这可能对结果产生一定影响。其次，文献检索仅限于中英文，未涵盖其他语言的研究，这可能影响到文献的全面性和研究结果的外部效度。最后，由于不同研究使用的网络成瘾评估工具不尽相同，存在一定的测量工具异质性，为此本研究通过标准化均数差合并效应量的方法，确保了各结局变量的可比性，尽量减少了测量工具差异带来的影响。

未来研究可以在控制饮食和日常活动的基础上，进一步探索身心运动疗法对网络成瘾的长期效果。此外，扩大文献检索范围，涵盖其他语言的研究，将有助于提高研究的全面性和外部效度。更高质量的多中心RCT研究将为制定更为科学、系统的干预策略提供更为坚实的证据。

**4 结论**

当前证据表明，体育舞蹈、太极拳、八段锦改善网络成瘾症状效果更好，极具推广价值。

**作者贡献：**

**利益冲突生命：**本研究不存在任何利益冲突。

**参考文献**

1. 刘婷婷,任丽英,杨莹.青少年网络成瘾研究进展[J].中国药物依赖性杂志,2015,24(05):396-400.DOI:10.13936/j.cnki.cjdd1992.2015.05.015.
2. 阳杨,朱天民,黄冰洁,等.互联网成瘾综合征的干预疗法概述[J].辽宁中医杂志,2013,40(07):1497-1500.DOI:10.13192/j.ljtcm.2013.07.223.yangy.092.
3. JORGENSON A G,HSIAO C J,YEN C F.Internet addiction and other behavioral addictions[J]. Child Adolesc Psychiatr Clin North Am,2016,25(3):509-520.
4. ZHER M,KHAN R B,SALIM M,et al.The relationship between Internet addiction and anxiety among students of University of Sargodha[J].Int J Human Soc Sci,2014,4(1):288-293.
5. Olson, J. A., Sandra, D. A., Colucci, ´E. S., Bikaii, A. A., Nahas, J., Chmoulevitch, D., Raz,A., & Veissi`ere, S. P. L. (2022). Smartphone addiction is increasing across the world:
6. A meta-analysis of 24 countries. Comput. Human Behav. 129, April 2022, Article No. 107138. 10.1016/j.chb.2021.107138.
7. Wang H, Li X, Lok G K I, et al. Family-based therapy for internet addiction among adolescents and young adults: A meta-analysis[J]. Journal of Behavioral Addictions, 2024.
8. Chen Y, Zhang L, Liu Y, et al. Acupuncture for Internet addiction: A protocol for systematic review[J]. Medicine, 2021, 100(12): e24872.
9. Ma C, Yan J, Hu H, et al. Associations between 24-h movement behavior and internet addiction in adolescents: a cross-sectional study[J]. International Journal of Environmental Research and Public Health, 2022, 19(24): 16873.
10. Chen H, Xiao M, Lin Y, et al. Current development of a nonpharmacological intervention approach for mild cognitive impairment patients and a clinical trial in China[J]. Journal of Translational Internal Medicine, 2022, 10(1): 5-8.
11. Chen H, Dong G, Li K. Overview on brain function enhancement of Internet addicts through exercise intervention: based on reward-execution-decision cycle. Front Psychiatry. 2023;14:1094583.
12. Yang H, Guo H, Zhu Z, et al. Intervention of Internet Addiction and Smartphone Addiction: an Umbrella Review of Systematic Reviews and Meta-Analyses[J]. Current Addiction Reports, 2024, 11(1): 125-148.
13. NCCIH. Complementary, Alternative, or Integrative Health: What’s In a Name? (2018). Available online at: https://www.nccih.nih.gov/health/ complementary-alternative-or-integrative-health-whats-in-a-name
14. Siu P M, Angus P Y, Tam B T, et al. Effects of Tai Chi or exercise on sleep in older adults with insomnia: a randomized clinical trial[J]. JAMA network open, 2021, 4(2): e2037199-e2037199.
15. Guo C, Xiang G, Xie L, et al. Effects of Tai Chi training on the physical and mental health status in patients with chronic obstructive pulmonary disease: a systematic review and meta-analysis[J]. Journal of thoracic disease, 2020, 12(3): 504.
16. Li Z , Liu S , Wang L , et al. Mind–Body Exercise for Anxiety and Depression in COPD Patients: A Systematic Review and META-Analysis[J]. International Journal of Environmental Research and Public Health, 2020, 17(1).
17. Zou L, Yeung A, Li C, et al. Effects of mind–body movements on balance function in stroke survivors: A mETA-analysis of randomized controlled trials[J]. International journal of environmental research and public health, 2018, 15(6): 1292.
18. Zou L, Yeung A, Zeng N, et al. Effects of mind-body exercises for mood and functional capabilities in patients with stroke: An analytical review of randomized controlled trials[J]. International journal of environmental research and public health, 2018, 15(4): 721.
19. Wang K, Liu S, Kong Z, et al. Mind-body exercise (Wuqinxi) for patients with chronic obstructive pulmonary disease: a systematic review and mETA-analysis of randomized controlled trials[J]. International Journal of Environmental Research and Public Health, 2019, 16(1): 72.
20. Zou L, Loprinzi P D, Yeung A S, et al. The beneficial effects of mind-body exercises for people with mild cognitive impairment: a systematic review with mETA-analysis[J]. Archives of physical medicine and rehabilitation, 2019, 100(8): 1556-1573.
21. Zhou S , Zhang Y , Kong Z , et al. The Effects of Tai Chi on Markers of Atherosclerosis, Lower-limb Physical Function, and Cognitive Ability in Adults Aged Over 60: A Randomized Controlled Trial[J]. International Journal of Environmental Research and Public Health, 2019, 16(5).
22. Zou L, Sasaki JE, Wei GX,et al. Effects of Mind⁻Body Exercises (Tai Chi/Yoga) on Heart Rate Variability Parameters and Perceived Stress: A Systematic Review with META-Analysis of Randomized Controlled Trials. J Clin Med. 2018 Oct 31;7(11):404.
23. Pirwani N, Szabo A. Could physical activity alleviate smartphone addiction in university students? A systematic literature review[J]. Preventive Medicine Reports, 2024: 102744.
24. 张天嵩. Stata软件network组命令在网状META分析中的应用[J]. 中国循证医学杂志, 2015, 15(11):5.
25. Liberati A, Altman D G, Tetzlaff J, et al. The PRISMA statement for reporting systematic reviews and mETA-analyses of studies that evaluate health care interventions: explanation and elaboration[J]. Journal of clinical epidemiology, 2009, 62(10): e1-e34.
26. 杨翠英,曾国凡.太极拳锻炼对大学生网络成瘾的影响[J].中国学校卫生,2017,38(02):292-294.DOI:10.16835/j.cnki.1000-9817.2017.02.041.
27. Zhang X, Yang H, Zhang K, et al. Effects of exercise or tai chi on Internet addiction in college students and the potential role of gut microbiota: A randomized controlled trial[J]. Journal of affective disorders, 2023, 327: 404-415.
28. **ao T, Jiao C, Yao J, et al. Effects of basketball and Baduan** exercise interventions on problematic smartphone use and mental health among college students: a randomized controlled trial[J]. Evidence‐Based Complementary and Alternative Medicine, 2021, 2021(1): 8880716.
29. Zhang K, Guo H, Zhang X, et al. Effects of aerobic exercise or Tai Chi Chuan interventions on problematic mobile phone use and the potential role of intestinal flora: a multi-arm randomized controlled trial[J]. Journal of Psychiatric Research, 2024, 170: 394-407.
30. Lan Y, Ding J E, Li W, et al. A pilot study of a group mindfulness-based cognitive-behavioral intervention for smartphone addiction among university students[J]. Journal of behavioral addictions, 2018, 7(4): 1171-1176.
31. 任建华,敖祖辉,贺春林,等.大学生网络成瘾的体育运动干预实验研究[J].黑龙江医药科学,2014,37(04):93-94.
32. 解飞.护生手机成瘾现状及八段锦健康教育干预研究[D].山西医科大学,2019.
33. 张缘.大学生手机依赖与体质健康的关系及运动干预研究[D].赣南师范大学,2018.
34. 刘诗洁,刘亚女,王林.运动干预和团体认知疗法对大学生手机依赖的实证研究[J].中国学校卫生,2022,43(06):825-829.DOI:10.16835/j.cnki.1000-9817.2022.06.007.
35. 李毅.正念训练对手机成瘾大学生反应抑制的影响[D].贵州医科大学,2022.DOI:10.27045/d.cnki.ggyyc.2022.000397.
36. Liu X, Clark J, Siskind D, et al. A systematic review and meta-analysis of the effects of Qigong and Tai Chi for depressive symptoms[J]. Complementary Therapies in Medicine, 2015, 23(4): 516-534.
37. Luo S, Mei Z, Fang G, et al. Effects of mind–body therapies on depression among adolescents: a systematic review and network meta-analysis[J]. Frontiers in public health, 2024, 12: 1431062.
